# A calibrated measure to compare fluctuations of different entities across timescales

**DOI:** 10.1038/s41598-020-77660-4

**Published:** 2020-11-26

**Authors:** Jan Chołoniewski, Julian Sienkiewicz, Naum Dretnik, Gregor Leban, Mike Thelwall, Janusz A. Hołyst

**Affiliations:** 1 Center of Excellence for Complex Systems Research, Faculty of Physics, Warsaw University of Technology, Koszykowa 75, 00-662 Warsaw, Poland; 2 Slovenian Press Agency, Tivolska 48, 1000 Ljubljana, Slovenia; 3 Artificial Intelligence Laboratory, Jožef Stefan Institute, Jamova 39, 1000 Ljubljana, Slovenia; 4 School of Mathematics and Computing, University of Wolverhampton, Wulfruna Street, Wolverhampton, WV1 1LY UK; 5 ITMO University, 49 Kronverkskiy av., Saint Petersburg, Russia 197101

**Keywords:** Nonlinear phenomena, Scientific data, Statistics

## Abstract

A common way to learn about a system’s properties is to analyze temporal fluctuations in associated variables. However, conclusions based on fluctuations from a single entity can be misleading when used without proper reference to other comparable entities or when examined only on one timescale. Here we introduce a method that uses predictions from a fluctuation scaling law as a benchmark for the observed standard deviations. Differences from the benchmark (residuals) are aggregated across multiple timescales using Principal Component Analysis to reduce data dimensionality. The first component score is a calibrated measure of fluctuations—the *reactivity* *RA* of a given entity. We apply our method to activity records from the media industry using data from the Event Registry news aggregator—over 32M articles on selected topics published by over 8000 news outlets. Our approach distinguishes between different news outlet reporting styles: high reactivity points to activity fluctuations larger than expected, reflecting a bursty reporting style, whereas low reactivity suggests a relatively stable reporting style. Combining our method with the political bias detector Media Bias/Fact Check we quantify the relative reporting styles for different topics of mainly US media sources grouped by political orientation. The results suggest that news outlets with a liberal bias tended to be the least reactive while conservative news outlets were the most reactive.

## Introduction

Fluctuations occur everywhere and are frequently sources of useful knowledge about intrinsic properties of systems, with diverse applications. When thermal activity^1^ or heart rate variability are measured^2^, they can give meaningful information if system-specific features are taken into account, such as the fluctuation–dissipation theorem^3^, or norms for heart rate variability in various groups of people^2,4^. In financial engineering, forecasting the volatility of stock prices^5,6^ is central to hedging strategies for stock portfolios^7^. There are multiple ways to quantify fluctuation levels, including the variance (or standard deviation), Fano factor^8^, and coefficient of variation^9^. Nevertheless, studying just absolute (variance) or relative (Fano factor) fluctuations can give misleading conclusions when the system’s size is ignored. For example, the variance $\sigma ^2_E$ of energy thermal fluctuations should be proportional to the number of particles in a system^3^ while for many non-thermal objects the variance in an ensemble of similar units (e.g. crop volumes in groups of fields) scales with the system size in agreement with Taylor’s law^10^. For time-dependent systems the rescaled range of an observable can depend on the length of the observation window $\Delta$ according to Hurst’s law^11^. In many cases time series fluctuation patterns can change with amplitude levels and in these systems generalized Hurst exponents should be calculated with Multifractal Detrended Fluctutions Analysis^6,12^.

In this study we introduce the notion of *reactivity* (*RA*), which is related to the residuals of the temporal fluctuation scaling law (or the temporal Taylor power law; TFS)^13,14^ that links the mean and the variance of a dynamical process for each observed entity through a power law. In this context, by a residual we mean the measured standard deviation of an observed system variable (henceforth called *activity*) calibrated against values expected from the TFS at a given timescale. The TFS is ubiquitous in complex systems^15^. Analyses of the fitted power law exponent have been applied e.g. to measure the lexical richness of texts in the presence of topical variations^16^, to characterize UK crime^17^ and human behavior under high pressure^18^, and to detect strong emotions in physiological timeseries^19^.

**Figure 1:**
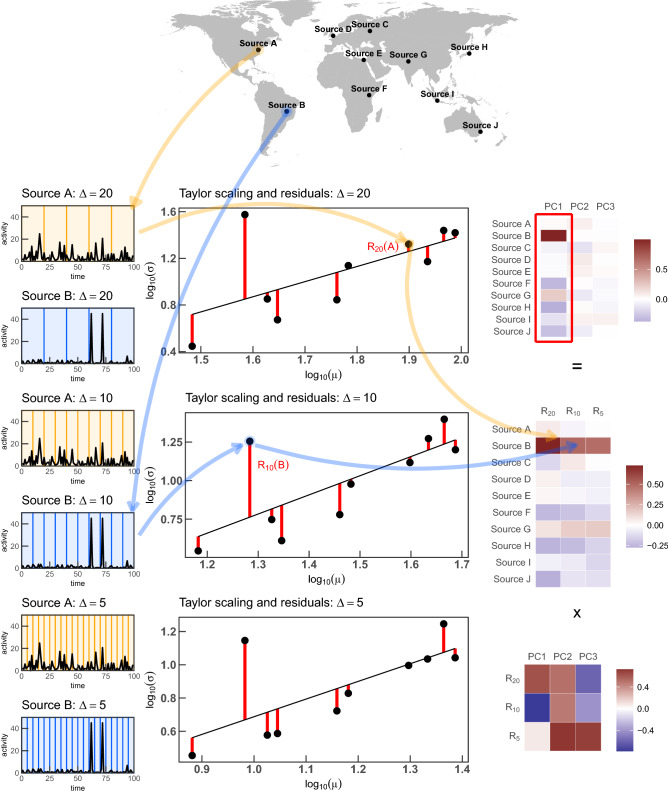
A scheme and a pipeline of methods used in this study. In this example we use 10 hypothetical **sources** (i.e., news outlets): (**A**)–(**J**) (top row on the plot) all writing about a given **concept** *k* (e.g., “European Union”); each is described with one timeseries reflecting their activity $f_{s,\Delta }^{(t)}(k)$, i.e., number of published articles over time (examples shown in the left column for sources A and B). Each series can be divided into **windows of size** $\Delta$ (here $\Delta _1=20$, $\Delta _2=10$, $\Delta _3=5$—see left column). In each window the number of articles is summed and then the **mean** $\mu$ and **standard deviation** $\sigma$ of these sums are calculated. These pairs for each source and window size are shown on the plot in the middle column and the fit to them in a log-log scale (solid line) is **the temporal fluctuation scaling** (TFS, Eq. (9)). Differences between $\sigma$ and TFS, given by Eq. (1) are **residuals** and are gathered in a matrix $\hat{\mathbf {R}}$ (middle plot in the right column) which is then an input for PCA. As an outcome of PCA we obtain a matrix $\hat{\mathbf {P}}$ of projections to new dimensions—Principal Components (PCs, top plot in the right column) and a transformation matrix $\hat{{\mathbf {G}}}$ (bottom plot). The **first PC**, i.e. the value in *s*-th row of the first column of the matrix $\hat{\mathbf {P}}$ is the key observable in our study and we will use the **reactivity** $RA_s(k)$ for the keyword *k* and source *s*. World map has been obtained using ggplot2 **R** package^20^ using maps **R** package data^21^.

Differences between entities in the above-mentioned datasets may cause them not to follow the TFS strictly. Although fluctuation scaling residuals cluster in geographical and financial data^15^, properties of residuals seem to have been rarely studied. One somewhat similar application of the TFS is date abnormality quantification by measuring the fluctuation scaling of adjective usage in Japanese blogs^22^ but this focused on detecting special dates rather than quantifying activity fluctuations.

The concept of reactivity will be applied to data from the Event Registry news aggregator^23^, containing over 32M articles on 39 topics (we will also call them *keywords* or *concepts*, interchangeably) published by over 8000 news outlets (also *sources*, *publishers*). Understanding media activity is a challenge because of the need to take into account differences in size, scope and political bias, as well as differences in reporting styles by topic and multiple timescales. Due to the complex nature of online news media (many interacting units participating in dynamic information exchanges), it is unsurprising that the activity of news outlets, measured by the number of articles published over time, follows the TFS^24^.

In the paper, we study fluctuations in news outlet activities $f_{s,\Delta }^{(t)}(k)$, focusing on multiple topics *k* (e.g. *Climate change* or *European Union*) and timescales $\Delta$ (from a few minutes to a few months). We found that the TFS residual value $R_{s,\Delta }(k)$ for a given news outlet *s* is more characteristic of a timescale $\Delta$ than of a topic *k*. To aggregate properties of fluctuations at different timescales, for each concept *k* we considered the 14-dimensional space corresponding to corrections to TFS for $\Delta \in \left\{ 1\,{{\mathrm {min}}},\dotsc ,60\,{{\mathrm {days}}}\right\}$ and performed Principal Component Analysis to reduce the dimensionality of the data set. The first component score is our calibrated measure of fluctuations: the *reactivity* $RA_s(k)$ of publisher *s* when reporting a topic *k*, see Eq. (3). A negative reactivity means smaller-than-typical fluctuations, and a positive reactivity means larger-than-typical fluctuations. We found that the median reactivity of news outlets from a given country was high if significant happenings related to a topic had taken place during the period analyzed. Additionally, we considered news outlet reactivity by political bias provided by mediabiasfactcheck.com. Analyses showed that news outlets with a liberal bias were, on average, less reactive while conservative news outlets were more reactive.

We use a pipeline of multiple theoretical approaches. The simplified scheme in Fig. 1 summarizes the fundamental concepts of this study for later reference.

## Results

Our reactivity method is described with news publishing data from the Event Registry (ER) dataset^23^. We extracted all articles published in 2018 mentioning one of ${\mathscr {K}}$ topics ($K=|{\mathscr {K}}|=39$, for details see Dataset info subsection of Methods). We divided the news articles into sets corresponding to topics ${\mathscr {K}}$ and publishers ${\mathscr {S}}$ ($|\mathscr {S}|=8155$), obtaining a list of article publication dates and times $f_s(k)=\{\tau _{s,1}(k), \tau _{s,2}(k), \dotsc \}$ for each keyword *k* and publisher *s*.

**Figure 2:**
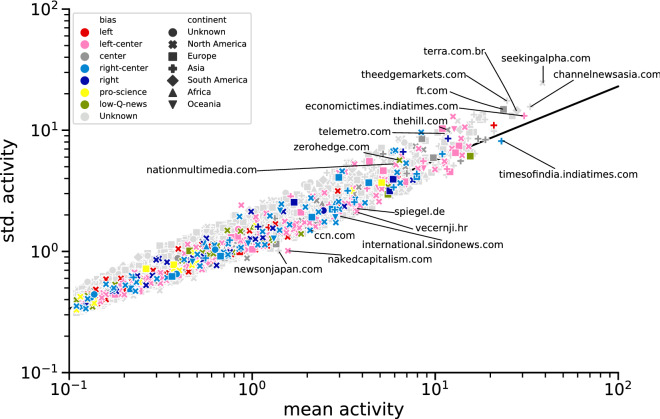
An example temporal fluctuation scaling plot for the keyword $k=$ China ($\Delta$=1 day). Each point represents one publisher *s*, its mean $\mu _{s,\Delta }(k)$ and standard deviation $\sigma _{s,\Delta }(k)$ of activity timeseries calculated for time window size $\Delta$. The black line represents an OLS fit to the points after calculating the logarithm of $\mu$ and $\sigma$. The vertical distance from the line to a point for logarithmic variables is the residual $R_{s,\Delta }(k)$ as described in the “Results” section. Point colors represent source biases; shapes—sources’ continents. Several sources with known and unknown political bias are annotated.

The activity timeseries $f_{s,\Delta }^{(t)}(k)$ were obtained by aggregating a list of article publication occurrences with timestamps using $D=14$ time window sizes $\Delta \in \{1\,{\mathrm {min}}, \dotsc , 60\,{\mathrm {days}}\}$ for each of the 39 keywords separately (see Extracting timeseries from lists of articles subsection of Methods: Statistical Methods). Figure 1 visualizes the whole process for 1 keyword, 2 publishers and 3 time windows. This is shown in the left column. Next, the timeseries were processed using the TFS procedure (see Temporal fluctuation scaling subsection of Methods: Statistical Methods) to obtain the scaling exponent $\alpha _\Delta (k)$ and the multiplicative factor $B_\Delta (k)$. An example TFS plot is in Fig. 2, corresponding to the middle column of Fig. 1. We then calculated TFS residuals $R_{s,\Delta }(k)$ for each keyword *k* and each relevant combination of publisher *s* and time window size $\Delta$ as a logarithm of a ratio of observed source’s activity standard deviation $\sigma _{s,\Delta }(k)$ and an expected source’s activity standard deviation $\tilde{\sigma }_{s,\Delta }(k)$. The last value results from the mean activity $\mu _{s,\Delta }(k)=\left\langle f_{s,\Delta }^{(t)}\right\rangle$ and the TFS law, i.e.

1 $$\begin{aligned} R_{s,\Delta } = \log _{10}\left[ \frac{\sigma _{s,\Delta }}{\tilde{\sigma }_{s,\Delta }}\right] \text {, where } \tilde{\sigma }_{s,\Delta } =B_\Delta \left\langle f_{s,\Delta }^{(t)}\right\rangle ^{\alpha _\Delta } \end{aligned}$$

For each concept *k* we then have a $S\times D$-dimensional matrix $\hat{\mathbf {R}}(k)$ containing TFS residuals $R_{s,\Delta }(k)$, i.e., logarithmic differences between observed activity fluctuations of a publisher *s* in a given timescale $\Delta$ and corresponding predictions resulting from the TFS scaling law that contains information about the magnitudes of fluctuations of other publishers.

The residuals $R_{s,\Delta }(k)$ are shown as red segments in the middle column of Fig. 1 and corresponding cells in matrix $\hat{\mathbf {R}}$ in the middle panel of the right column in the same plot.

### Correlations of residuals across timescales and concepts

Let us now investigate whether the TFS residuals are similar for a given publisher across all concepts *k* and time window sizes $\Delta$. For this, we calculate the Pearson correlation between residual values obtained for every pair of $(k,\Delta )$ combinations, a total of $N=K\times D=546$ combinations. The aggregation was performed over all sources publishing on both topics—see the Agglomerative Clustering of correlation matrices subsection of Methods for details.

**Figure 3:**
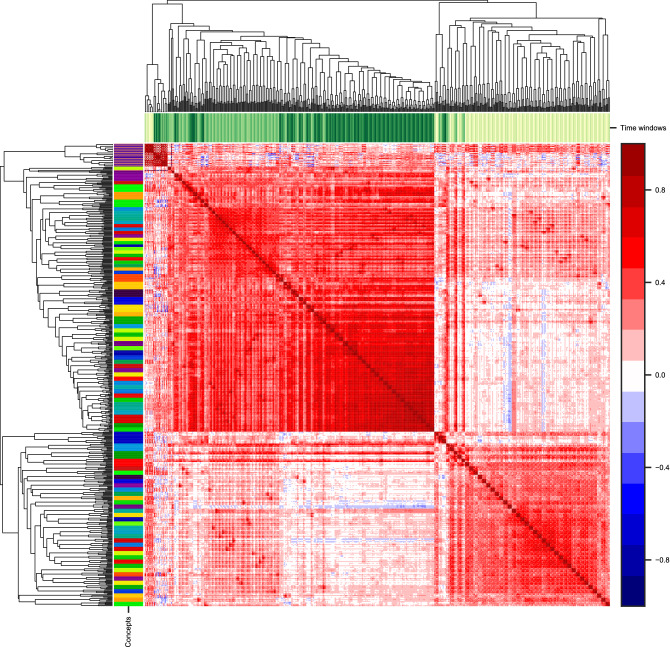
Agglomerative Clustering of a correlation matrix of residuals for all concepts and time window sizes. Each row/column stands for one of 546 $(k, \Delta )$ combinations. A color bar for the correlation matrix values is on the right hand side. Additional color labels on top of the matrix represent time window size $\Delta$ (lightest—1 min, darkest—60 days); color labels on the left side of the matrix represent different keywords *k*. Residuals for short ($\Delta<$ 1 h), medium (1 h $<\Delta<$ 1 day), and long ($\Delta>$ 1 day) time windows are clustered together; inside the three clusters, residuals for most keywords *k* tend to be close to each other.

Figure 3 gives a $546\times 546$ matrix of correlations $\rho (k_1,\Delta _1;k_2,\Delta _2)$ between all possible combinations of $(k,\Delta )$. Each row/column stands for one concept-time window size combination. The columns/rows were clustered using Agglomerative Clustering and their order is from the leaves of the resulting dendrogram. The most striking observation is that around 92% of the elements of this matrix are positive valued and the mean value of off-diagonal elements is 0.26. Moreover, the residuals separate roughly into three groups connected to different timescales—$\Delta \le 1\,{\mathrm {h}}$ (*short*), $1\,{\mathrm {h}}<\Delta \le 1\,{\mathrm {day}}$ (*medium*), $\Delta > 1\,{\mathrm {day}}$ (*long*). Moreover, inside the groups residuals for a keyword also tend to be adjacent—to see this, compare the sizes of the color clusters and the size of the thinnest color marker. To further quantify this observation, we calculated the means of in-group and between-group correlation matrix elements for the groups (see Supplementary Information: Aggregated correlation matrices). Averaging over all concepts reveals dependencies between $R_{s,\Delta }(k)$ for various pairs of time window sizes $\Delta$. The *long* group had high in-group correlations (0.55) compared to *medium* (0.38) and *short* (0.36); residuals in the *medium* group positively correlated with the *long* group (0.33) and slightly less to the *short* group (0.16). There was no correlation between *short* and *long* timescales (0.06). Similarly, we averaged the correlation matrix elements for pairs of concepts over all time window sizes. The mean of the diagonal elements was 0.52, and the mean of the off-diagonal elements was 0.26.

Thus, residuals for the given source and concept positively correlate for time windows of similar size. In the same manner, residuals for a given source and time window positively correlate across different keywords. This is surprising because the residuals for each concept were calculated using different article sets.

### Principal components analysis of publisher residuals for each concept

So far we have considered standard deviation residuals separately for different lengths of observation windows $\Delta$. For every publisher *s* and for every topic *k* there are 14 standard deviation residuals $R_{s,\Delta }(k)$ in matrix $\hat{\mathbf {R}}(k)$ (the middle matrix in the right row of Fig. 1). To reduce the dimensionality of these observations we performed Principal Component Analysis (PCA) on the residuals $R_{s,\Delta }(k)$ assuming that values for different $\Delta$ are related to mutually orthogonal directions. PCA produces a matrix of projections of the original variables to Principal Components (PCs) $\hat{\mathbf {P}}(k)$ (top matrix in the right column) as well as a matrix $\hat{{\mathbf {G}}}(k)$ (bottom matrix) of new basis vectors that transforms $\hat{\mathbf {R}}(k)$ into $\hat{\mathbf {P}}(k)$ according to

2 $$\begin{aligned} \hat{\mathbf {P}}(k) = \hat{\mathbf {R}}(k) \hat{{\mathbf {G}}}(k) \end{aligned}$$

**Figure 4:**
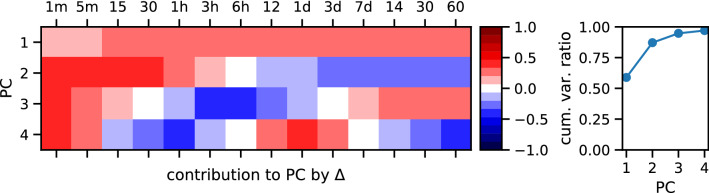
Principal Component Analysis of residuals by time window size for the keyword ***European Union*** **(clean data).** (left) Contributions of residuals $R_{s,\Delta }(k)$ for the $\Delta$ analyzed to the first four PCs (first four rows of matrix $\hat{{\mathbf {G}}}$). The first principal component is roughly the arithmetic mean of the residuals over different timescales, the second PC has opposite loadings for long and short timescales. (right) A cumulative explained variance ratio for first four PCs. The first four PCs explain typically around 97% of variance.

The columns of the transformation matrix $\hat{{\mathbf {G}}}(k)$ are eigenvectors of the empirical covariance matrix $\hat{\mathbf {R}}(k) \hat{\mathbf {R}}^T(k)$ of residuals. The first column ${\mathbf {G}}^1(k)$ corresponds to the largest eigenvalue and it defines the direction of the first principal component axis^25^. To make the PCs comparable across keywords, we forced the directions of the axes to have the sign of the contribution from the shortest time window residual ($1\,{\mathrm {min}}$). The first PC (PC1, elements of the first column of the matrix $\hat{\mathbf {P}}(k)$) is almost the mean of the residuals for all time window sizes, i.e., all $\Delta$ contribute to this PC almost in the same way (see top row in the matrix on the left panel of Fig. 4). This usually explains around 50% of the variance (right panel in Fig. 4). The second PC (PC2, the second column of $\hat{\mathbf {P}}(k)$; 30% of variance) has opposing signs for contributions from residuals for time window sizes below 1 day and over 1 day; contributions for the extreme values were the highest. In the third PC (around 10% of variance), contributions for $\Delta \le 30\, {\mathrm {min}}$ and $\Delta \ge 3\,{\mathrm {days}}$ have opposite signs to those for $\Delta$ over 1 h and below 3 days. PC1 is the residual of a source for a given keyword averaged over the timescales analyzed. Thus, high absolute values of PC1 indicate atypically low/high fluctuations in timeseries of the source for one or more time window sizes. PC2 shows whether the atypical fluctuations were observed for short or long timescales. PC3 seems to describe the variance of a source in scales of several hours; it is probably connected to whether a source has a day/night activity cycle. PCs in the cleaned dataset (Fig. 4) have a very similar composition to that in the raw data (see Supplementary Information: Cleaning dataset).

We calculated a correlation matrix of vectors representing each source’s selected PC *p* for each keyword *k*, as for the residuals.

**Figure 5:**
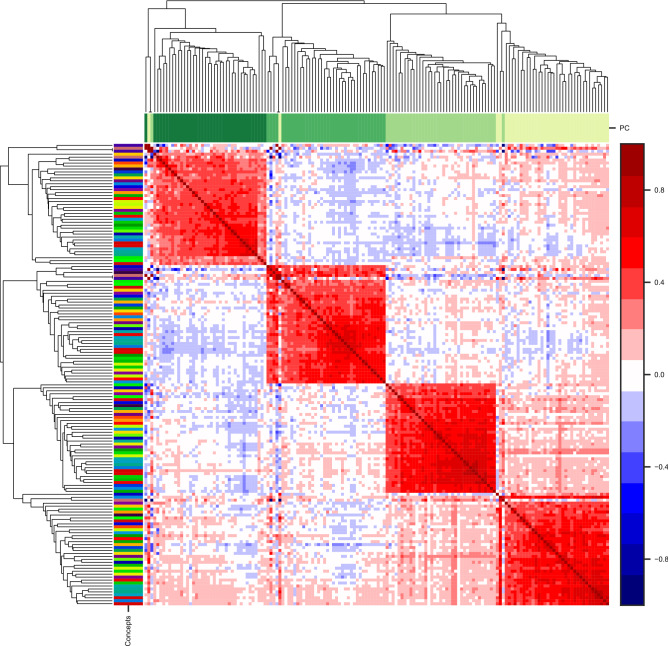
Agglomerative Clustering of a correlation matrix of the first four principal components of the residuals $P_{s,p}(k)$ ($a=1,\dotsc ,4$) for all concepts *k*. Each row/column stands for one (*k*, *p*) combination. A color bar for the correlation matrix values is on the right hand side. Additional color labels above the matrix represent an order of PC *a* (lightest—1st PC, darkest—4th PC); color labels on the left side of the matrix represent different keywords *k*. PCs $P_{s,p}(k)$ of the same order *p* are clustered; the mean correlation coefficient $\left\langle \rho \left( k_1,p_1;k_2,p_2)\right) \right\rangle _{p_1=p_2}=0.44$.

Figure 5 shows that for each PC value, all keywords form a separate cluster. The mean correlation inside a cluster corresponding to results for one PC and all keywords was 0.44.

This finding means that the reporting style is intrinsic for a source rather than a keyword, nevertheless, we believe that slight differences in the values for various keywords might capture subtle reporting style differences between groups of sources when aggregated. We select the first PC as the main indicator of source *s* reporting style on a given topic *k*—*reactivity* $RA_s(k)$—as the first principal component score:

3 $$\begin{aligned} RA_s(k) \equiv P_{s,1}(k)= \mathbf {R}_s(k) {\mathbf {G}}^1(k) \end{aligned}$$

where $\mathbf {R}_s(k)$ is the *s*-th row of matrix $\hat{\mathbf {R}}(k)$ and ${\mathbf {G}}^1(k)$ denotes the first column of transformation matrix $\hat{{\mathbf {G}}}(k)$.

For news outlet activities, an intuitive interpretation would be that higher-than-expected fluctuations show a *reactive* reporting style for a given topic by a given source (e.g. activity influenced by external events) and lower fluctuations indicate a *stable* reporting style. These differences are subtle but clearly visible in the Supplementary Information: Additional dataset info. A source’s relative burstiness for a topic compared to other news outlets might be useful when interpreting an article as a signal lateral to general interest (number of articles) or sentiment towards the topic.

### Aggregated reactivity

Having defined a measure of the reactivity $RA_s(k)$ of publisher *s* towards topic *k*, we used it to quantify the typical reporting style of groups of publishers. Two features of news outlets that we have used for aggregation are their *political bias* and *country of origin*. Below, we analyze patterns of median reactivity by group.

#### Reactivity by country

**Figure 6:**
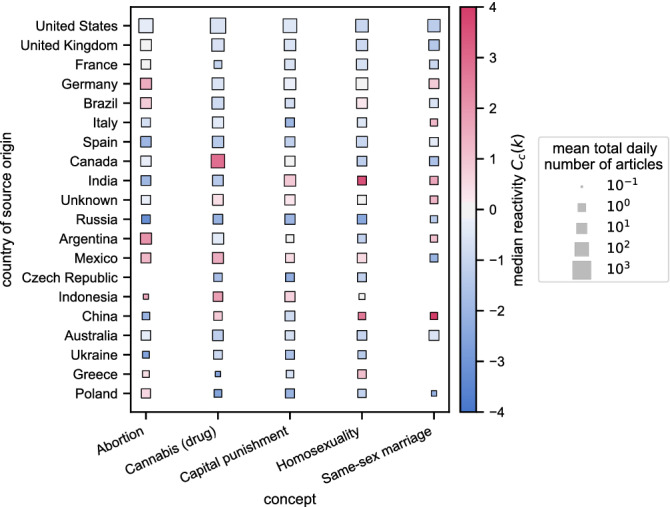
The median reactivity $C_c(k)$ of the reactivity for all sources from a given country *c* for keywords *k* related to “polarizing” concepts. Square size is proportional to the mean daily number of articles published by sources from a given country *c* on a given topic *k*; color represents the median reactivity $C_c(k)=\langle {RA}_{s}(k)\rangle _{s\in \mathscr {S}_c}$ of sources from the country *c*. Missing squares indicate that there were no publishers from the country that published at least 36 articles on the topic in our dataset. Red symbols correspond to topics that were reactively discussed in the country in 2018.

The median $RA_s(k)$ by country for polarizing concepts (see the Concepts subsection of Methods) are in Fig. 6 (for other concepts—see Supplementary Information: Aggregated reactivity). Rows represent countries with the most sources tracked by ER with the highest topping the list; columns stand for concepts. Colors indicate the medians $\langle {RA}_{s}(k)\rangle _{s\in \mathscr {S}_c}=C_c(k)$ of the reactivity for all sources $s\in \mathscr {S}_c$ from a given country *c* when reporting a given topic *k*. Recall that reactivity $RA_s(k)$ has a logarithmic nature (since the original variables were logarithmic), so one unit difference translates to an order of magnitude (factor 10) difference in fluctuations. It is clear that sources from United States, United Kingdom, France, and Australia have fewer-than-typical fluctuations for all the polarizing concepts—colors are very close to white ($C_c(k)\approx -0.7$). Russian, Czech, Polish, and Ukrainian sources also seem to typically cover the topics stably ($C_c(k)\approx -1.5$), Chinese, and Indonesian—reactively ($C_c(k)\approx 1.0$). German and Argentinian publishers most reactively reported on *Abortion* ($C_c(k)\approx 1.5, 2.1$, respectively); Canada and Indonesia on *Cannabis* (2.9, 1.8); India and Indonesia on *Capital punishment* (0.9, 0.7); India and China on *Homosexuality* (3.6, 2.7); China and India on *Same-sex marriage* (5.2, 1.6). Interestingly, in Italy the topic *Homosexuality* was reported rather stably ($-0.5$) but *Same-sex marriage*—reactively (1.2). The most notable stable reporting was observed for Russian, Chinese, Spanish and Indian sources for *Abortion* ($-3.2, -2.1, -2.0, -1.9$). Stable reporting was also found for *Cannabis* by Polish and Russian outlets ($-2.5, -2.11$); *Capital punishment* in Czech Republic and Poland ($-2.2, -2.0$); *Homosexuality* in Russia, Ukraine and Canada ($-2.4, -1.3, -1.2$), *Same-sex marriage* in Poland and Mexico ($-2.2, -2.0$). The values seem to reflect the temperature of the national views towards a given concept. For example, in 2018, Canada legalized *Cannabis*^26^ and India legalized *Homosexuality*^27^. Publishers from both countries tended to report reactively on the respective topics, presumably driven by the legislative changes. Similarly, in China, there were two major rulings on *Same-sex marriages*^28^. Indonesia reactively reported (1.9, see SI) on *Terrorism* as it suffered six terrorist attacks in 2018^29^; the high reactivity of Italian media for *Shooting* (4.1, see SI) might be an effect of the Macerata shooting^30^. The French media were reactive towards *China* (3.6, see SI) as 2018 was an important year for bilateral relations between the two countries^31^. Recall that high *reactivity* does not necessary mean that the given keyword was frequently discussed in the given country. In fact Fig. 6 indicates that *Same-sex marriage* and *Homosexuality * were discussed in China very reactively but less frequently than *Capital punishment*, which was reported very stably (compare square colors and sizes in the above-mentioned figure). Similarly, *Homosexuality* in India was a reactive topic although it attracted less interest than *Abortion* or *Cannabis*, which were reported very stably. More such examples can be found in the Supplementary Information.

#### Reactivity by political bias

The above analysis has shown that the median value of $\langle {RA}_{s}(k)\rangle _{s\in \mathscr {G}}$ reflects the general attitude of a group of sources $\mathscr {G}$ (e.g. from the same country) towards a topic *k*. Around 10% of the publishers in Event Registry were also annotated by mediabiasfactcheck.com for political bias—see the Dataset info: Political bias of sources subsection of Methods. This is used to investigate the reactivity of sources grouped by political bias.

A general tendency was that sources marked as *left* represent (on average) a stable reporting style towards most concepts, while *right* tended to have a reactive/bursty style. For 30 out 39 analyzed concepts (see Supplementary Information: Aggregated reactivity), sources in the *right* group had the highest or the second highest reactivity, while the *left* group had the lowest or the second lowest for 26 concepts; often (in 18 cases) all the groups *left-center, center, right-center* had their median above the *left* and below the *right*. The *right* was relatively nonreactive for the concepts *Kim Kardashian*, *Marvel comics*, and *Same-sex marriage*; the *left* was relatively reactive for *Cannabis (drug)*, *Climate change*, and *Shooting*. The positions of *pro-science* and *low-Q-news* (ie. *low quality news*) did not have a stable pattern, perhaps caused by the low number of sources in these groups. The sources with annotated bias were mostly of US origin.

To compare reactivity values across all of the analyzed keywords, we calculated the median reactivity of each bias group *b* for each keyword *k* as $\langle {RA}_{s}(k)\rangle _{bias(s)=b}=P_b(k)$, where *s*—publisher, *bias*(*s*)—a political bias of a source *s*. We defined the relative median reactivity of a bias group for a keyword as follows:

4 $$\begin{aligned} \tilde{B}_b(k)=B_b(k) - \min _{b'} B_{b'}(k) \end{aligned}$$

**Figure 7:**
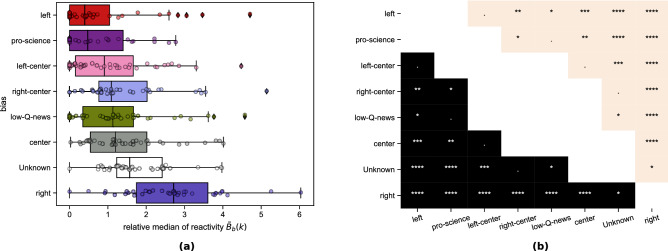
Comparison of relative median reactivity $$\tilde{B}_b(k)$$ between political bias groups for all keywords. *Left*-oriented sources are generally less reactive than the *right*-oriented sources. (**a**) values of relative median reactivity $$\tilde{B}_b(k)$$ (see Eq. (4)) for all keywords by political bias. (**b**) Results of pairwise Dunn median tests with a two-step Benjamini–Krieger–Yekutieli FDR adjustment^32^; the adjusted *p*-values describe the likelihood of the observed difference in medians of two samples assuming there is no difference between the medians of their populations. Thus, the lower the *p*-value, the more statistically significant the difference between the two group medians. $$****-p\in [0,0.001)$$, $$***-p\in [0.001, 0.005)$$, $$**-p\in [0.005, 0.01)$$, $$*-p\in [0.01,.05)$$, $$.-p\in [0.05, 0.1$$), otherwise $$p\in [0.1,1]$$. The color indicates which group had the higher median—black if the median of the group from the corresponding row was higher than the median of the group from the corresponding column; white—the opposite case.

Thus, a bias group with the lowest $B_b(k)$ for a given *k* will indicate $\tilde{B}_b(k)=0$. In Fig. 7 (left panel), reports $\tilde{B}_b(k)$ by a bias group for all the keywords. To quantify the general tendencies of the relative median reactivity $\tilde{B}_b(k)$, we performed a Kruskal–Wallis test with the FDR-adjusted Dunn’s test for pairwise comparisons^32^ of the bias groups; the results are in Fig. 7 (right panel). Both *left* and *pro-science* typically had lower $\tilde{B}_b(k)$ values than the remaining groups but are statistically indistinguishable from each other. On the other hand, the relative reactivity $\tilde{B}_b(k)$ for *right* was significantly higher than the remaining groups; *Unknown* scored similarly but even this group had lower average reactivity than *right*. *Low-Q-news* and *left-center* were distinguishable from *right* and *Unknown* only; the relative median reactivity $\tilde{B}_b(k)$ of *center* and *right-center* are over *left* and below *right*. The statistical tests confirmed that the *left* is significantly less reactive than the *right*.

## Discussion

This paper reports a novel two-step method to quantify the temporal fluctuations of a variable $f_{s,\Delta }^{(t)}(k)$ describing the dynamics of entities *s* belonging to a complex system, such as news outlets in the media space. In this case $f_{s,\Delta }^{(t)}(k)$ is the number of articles published by the outlet *s* about a keyword *k* during a time window $\Delta$ around a time moment *t*. The first step consists of checking whether the Temporal Fluctuation Scaling law (TFS) is valid for the system of entities *s* and, if so, determining TFS residuals for various time window sizes $\Delta$. The residuals are ratios of empirical standard deviations $f_{s,\Delta }^{(t)}(k)$ (measured from a time series) to the value predicted by the TFS law^15,18^. The second step is dimensionality reduction using Principal Component Analysis (PCA) to obtain a value independent of the time window size. The First Principal Component *PC1  * contains aggregated information about fluctuations in all timescales and we call this *reactivity*. To give a practical application, we used a case study of the online news aggregator Event Registry^23^.

We started with calculating TFS for activity timeseries in several timescales $\Delta$ for each keyword *k* separately (Fig. 2) and then obtained TFS residuals $R_{s,\Delta }(k)$ (see Eq. (1)) in each timescale $\Delta$ for every source *s*. Using a correlation matrix of the residual values (Fig. 3), we showed that the residuals of a given source positively correlate across different keywords for similar time window sizes. Moreover, using this method there are three timescales for fluctuations of publishers’ activities (*short*—$\Delta \le$ 1 h, *medium*—1 h $<\Delta \le$ 1 day, *long*—1 day $<\Delta$) reported in^24^. We conclude that reporting style is intrinsic to a publisher but small differences in the values for keywords capture subtle differences in reporting style. A similar observation was reported in^15^ for a spatial clustering of world precipitation data and a clustering of financial stocks from the same sectors.

To extract the most substantial factors that could aggregate the residuals over different timescales we utilized PCA. PCA reveals linear combinations of residuals at different timescales to form an uncorrelated orthogonal basis set where consecutive PCA components correspond to the highest variances between news sources (Eq. (2)). From the results, PC1, later called reactivity $RA_s(k)$ (Eq. (3)), is a good summary of source *s* deviation from the typical variation of activity on a topic *k*. In fact, the reactivity value for the dataset has nearly the same loadings from residuals $R_{s,\Delta }(k)$ at each timescale $\Delta$. On the other hand, the PC2 has opposing loadings from residuals in short ($\Delta < 1$ day) and long ($\Delta > 1$ day) timescales. The 1st and 2nd PCs together explain about 80% of the cumulative variance for the dataset (Fig. 4) and therefore can be used to reduce the system dimensionality. A correlation matrix of the PC values $\hat{\mathbf {P}}(k)$ (Fig. 5) upholds our observation—PC values for a given source obtained from various keywords positively correlate and therefore seem to be intrinsic to the source.

We then aggregated reactivity values grouped by political bias or country of origin to assess the relative reporting style of the groups towards different topics. First, we showed that the reactivity measure can detect which topics were reported reactively by comparing the results grouped by source country (Fig. 6), e.g., legalization of Cannabis in Canada and Homosexuality in India. Such topics would not be highlighted and could be disregarded if one relied only on activity, i.e., taking into account only the number of articles about the given keyword. Then, we analyzed differences in reporting style between (mostly American) sources grouped by political bias (Fig. 7). Groups *left* and *pro-science* had been typically less reactive than others for the concepts. On the other hand, news outlets annotated as *right* were usually the most reactive.

The reactivity value is dimensionless and normalized which enables comparisons of source reactivity across different context. For example, it could be applied to compare the relative volatility of stocks in various stock exchanges. Although our *reactivity* $RA_s(k)$ coefficient (Eq. 3) is similar in concept to Fano factor^8^ and the coefficient of variation^9^ it has several advantages compared to them. (i) It is based on a comparison of activity fluctuations of a given object to activity fluctuations in an ensemble of other objects that obeying temporal fluctuation scaling, so it has a well-defined *zero level*. (ii) Its single value takes into account fluctuations at multiple timescales $\Delta$ due to Principal Component Analysis. (iii) It is not correlated ($\rho =-0.05$) with activity and so can be used to compare fluctuations in entities of different sizes. In fact, the correlation between the Fano factor and activity for the ER data set is about $\rho =0.3$. Thus, this factor overestimates fluctuations for large publishers and the correlation between the coefficient of variation and activity is about $\rho =-0.57$, overestimating fluctuations for small and medium outlets (for details—see Supplementary Information: Measure comparisons).

Another way of comparing reactivity to the Fano factor and the coefficient of variation is to use them instead of TFS residuals in the first step of our method and perform the second step and analyses without changes (see Supplementary Information: Measures comparisons). Correlation matrices obtained with these alternative variability measures could not be clustered into three timescales. For the Fano factor, two regimes were observed (for $\Delta$ below or above 15 min) and a typical correlation inside each cluster was lower than for the reactivity *RA*. For the coefficient of variation, two regimes also exist (for $\Delta$ below or above 1 day) and almost all values positively correlated. Our reactivity approach finds clustering in *three timescales* (Fig. 3) that cover both divisions found by the Fano (Supplementary Fig. S18) and coefficient of variation (Supplementary Fig. S17) approaches separately. It follows that while the Fano is sensitive to the short timescale division and the coefficient of variation to the long timescale, reactivity takes into account all the timescales in the case analyzed. Moreover, aggregating variability by the *political bias* of a source (Fig. 7) was most successful when reactivity *RA* was used, because ordering political biases by median relative variability was similar to ordering on a political spectrum—from *left* through *center* to *right*. For the coefficient of variation (Supplementary Fig. S23), the ordering seemed uninformative—the *left-center* group had the lowest relative fluctuations and *left* had the highest. For the Fano factor, only *left* biased sources had a median significantly different from other bias groups.

Our reactivity parameter, $RA_s(k)$ is related to fluctuations of a single unit *s* at different timescales but it should not be mistaken for the generalized Hurst *H*(*q*) exponent that is calculated from a scaling law describing *q*-th order fluctuation as the function of time window size $\Delta$. In fact, *reactivity* *RA* is related to the scaling behavior (TFS) that comes from comparing different units. This means that *reactivity* takes into account features following from a set of entities while the generalized Hurst exponent *H*(*q*) reflects only scaling for the timeseries of a single entity. The relation between the TFS scaling exponent and the Hurst exponent *H*(2) was discussed in^15^. Since the TFS scaling law takes into account fluctuations of order $q=2$, we are not considering how the *RA* measure is dependent on the multifractality of a timeseries when the Hurst exponent is *q*-dependent.

We have summarized above the properties of the reactivity measure *RA*, which is related to the first principal component PC1 of the residuals matrix $\hat{\mathbf {R}}(k)$. The first two PCs (PC1 and PC2) have also been used to detect corrupted time series (see SI: Cleaning Dataset) in our dataset. The first type of data corruption is related to an abnormally large number of published articles by a given outlet in a short time period (a few minutes to a few hours) and is likely to be result of changes in the website shape that misleads the web crawler. This can be detected by a high value of the PC1 for this publisher. The second type of data corruption is related to missing records in a period that can be caused by the lack of a functional crawler. This can be detected by a value of PC2 indicating large corrections to TFS in long timescales for this publisher.

We believe the new approach is universal and can be applied to other fields where a proper assessment of the relative amount of signal variation is critical (e.g. financial markets^33–35^, or neuroscience^36,37^). Additionally, the number of sources annotated by political bias was relatively low and skewed towards the US which significantly decreased the reliability and generality of the results, although giving a more homogeneous dataset to analyze. Unfortunately, mass-scale automatic bias classification with high accuracy seems out-of-reach for now^38,39^. Moreover, more information could be extracted from similar datasets by inspection of consecutive PCs. For example PC2 indicates whether the fluctuations described by PC1 are biased towards short or long timescales. A method to adjust the mean and variance of corrupted timeseries might be developed using the new method to compensate for the two described disturbances (artificial activity spike, periods of missing data). One of the biggest disadvantages of the new measure is its high computational complexity, but this could be resolved using approximate methods^40^.

In conclusion, we have introduced a method that uses predictions from a fluctuation scaling law to benchmark observed standard deviations and our reactivity measure *RA* aggregates information about fluctuations at different timescales. We show that the approach makes it possible to compare news outlet reporting styles by geography and political orientation.

## Methods

### Dataset info

Online news media are hard to analyze on a large scale due to their distributed character compared to social media. Social media data is concentrated around a few important platforms (like Twitter or Facebook) but the news industry has a huge number of news outlets. Nevertheless, mass-scale surveillance of the online news industry is possible with news aggregators such as Event Registry (ER, www.eventregistry.org). ER is an online system which collects, annotates, clusters, and stores news items from online sources around the globe^23^. The system has been maintained by the Artificial Intelligence Laboratory (Jožef Stefan Institute, Ljubljana, Slovenia) since 2014 and tracks tens of thousands of publishers in near real-time. While its purpose is to track world events by clustering news items (across different languages), access to this database was provided by one of the coauthors. We queried the system to download all articles containing one of selected concepts (see the “Concepts” subsection of “Materials and methods”). Each article was represented as a tuple: *publisher, keyword (topic), timestamp*.

#### Sources

When our data set was created there were 8155 sources in ER that published at least three articles per month about at least one of the analyzed concepts. To examine geographical and political differences between the publishers, we considered the sources’ countries of origin and political biases, where applicable. Source counts by continent can be found in Table 1.

**Table 1:** Sources by continents. Only sources which published at least one article per month about at least one of the analyzed keywords.

Continent	Sources	(With known bias)
Europe	3065	76
North America	2650	526
Asia	1015	71
South America	655	4
Africa	426	7
Unknown	190	7
Oceania	154	18

Source countries of origin are reported by ER. Sources from all continents were present in the dataset; over two thirds were in Europe (37%) or North America (33%). Asia (13%) and Africa (5%) are underrepresented in the set compared to their populations. The relatively small number of publishers from South America (8%) and Oceania (2%) seems to be justified by their relatively small populations. Geographic data was missing for 2% of sources.

#### Political bias of sources

Political bias data was gathered from Media Bias/Fact Check (MBFC, www.mediabiasfactcheck.com). At the moment of writing, the website listed 2896 online media bias annotations, but only 709 overlapped with our list. While its credibility is sometimes questioned, it has been regarded as accurate enough to be used as ground-truth for e.g. media bias classifiers^39,41,42^, fake news studies^43–45^, and automatic fact-checking systems^46–48^.

MBFC groups sources into 8 categories: *left* (92 sources in our list), *left-center* (255), *center* (147), *right-center* (125), *right* (53), *pro-science* (27), *fake-news* (21), *conspiracy* (17), *satire* (2). We merged the last three categories to one—*low-Q-news* (41). Following the website, sources marked as *left* are “moderately to strongly biased toward liberal causes through story selection and/or political affiliation”, *left-center* have “slight to moderate liberal bias”, sources marked as *right* are “moderately to strongly biased toward conservative causes” and *right-center* “media sources are slightly to moderately conservative in bias”. *Center* media are considered to have a minimal bias, provide factual and sourced reporting, and to be “the most credible media sources”. Sources with an extreme liberal/conservative bias are listed mainly as *fake-news* or *conspiracy*. Sources from all continents can be found in the MBFC list but the distribution is very US-centered—the vast majority of sources tracked by ER and annotated by MBFC are in North America (75%); the rest consist of a few European (10%), Asian (10%), Oceanian (4%), African (1.5%), and South American (1%) news outlets.

#### Concepts

One of natural language processing techniques implemented in ER is the extraction of concepts and entities (called here *topics* or *keywords*, despite often being longer than one word) involved in a given piece of news. ER identifies keywords using Wikipedia and so every keyword has an associated Wikipedia article. The set of concepts selected for the study consisted of 19 countries and 20 current topics. We chose a few countries from all the continents: North America (*United States*, *Mexico*), Europe (*United Kingdom*, *France*, *Germany*, *Austria*, *Poland*, *Slovenia*, and *European Union*), Asia (*China*, *India*, *Japan*, *North Korea*, *Iran*), South America (*Brazil*, *Argentina*), Africa (*Egypt*, *South Africa*, *Morocco*), and Oceania (*Indonesia*). The current topics set was divided into “polarizing” and “other” topics. The first group consists of concepts which we expect to be perceived in different way by various political groups (*Cannabis (drug)*, *Capital punishment*, *Abortion*, *Homosexuality*, *Same-sex marriage*). These concepts seem likely to differentiate between liberal and conservative views. For example, they were a vital part of questionnaire in the NetSense experiment^49–51^; unfortunately, Event Registry had too few articles on other topics used in the study (*Premarital sex*, *Euthanasia*) for statistical analyses. The “other” group contains keywords related to violence (*Terrorism*, *Shooting*), sports (*Real Madrid C.F.*, *Roger Federer*, *Usain Bolt*), celebrities (*Kim Kardashian*, *British royal family*), and other concepts covered nowadays (*Facebook*, *Islam*, *Climate change*, *Universe*, *Bitcoin*, *Blockchain*, *Marvel Comics*).

Numbers of sources and articles for each concept are in the Supplementary Information: Additional dataset info.

### Statistical methods

#### Extracting timeseries from lists of articles

The raw data for this study consists of lists of article publication dates and times $f_s(k)=\big \{\tau _{s,1}(k), \tau _{s,2}(k), \dotsc \big \}$ for each keyword *k* and publisher *s* that had at least three articles with the keyword. To obtain timeseries, we divided the observation period (01.01–31.12.2018, $T=1$ year) into non-overlapping windows of length $\Delta$ and counted occurrences of publications in window *t* as follows:

5 $$\begin{aligned} f_{s,\Delta }^{(t)}(k)= \Big |\big \{\tau _{s,i}(k) : t\Delta \le \tau _{s,i}(k)<(t+1)\Delta \big \}\Big | \end{aligned}$$

where $|\mathscr {X}|$ is the number of elements of a set $\mathscr {X}$. For a given $\Delta$, we have $W =\lceil T/\Delta \rceil$ time windows.

To allow statistical analyses, the longest period analyzed was 60 days. There was not much information in timescales below a few minutes as the exact position of the timestamp was often determined by the crawl time. We chose 14 time window sizes $\Delta \in \{$1 min, 5 min, 15 min, 30 min, 1 h, 3 h, 6 h, 12 h, 1 day, 3 days, 7 days, 14 days, 30 days, 60 days$\}$ for the study. When the window length $\Delta$ did not divide *T* without a reminder, we treated the last time window equally with the rest.

#### Temporal fluctuation scaling

The following introduces temporal fluctuation scaling (TFS), which was been applied to our data and served as a starting point to define TFS residuals.

First, we calculate means of the timeseries:

6 $$\begin{aligned} \left\langle f_{s,\Delta }^{(t)}(k)\right\rangle = W^{-1} \sum _{t=1}^{W}f_{s,\Delta }^{(t)}(k) \end{aligned}$$

then variances:

7 $$\begin{aligned} \sigma _{s,\Delta }^2(k)= \left\langle \left[ f_{s,\Delta }^{(t)}(k)\right] ^2\right\rangle - \left\langle f_{s,\Delta }^{(t)}(k)\right\rangle ^2 \end{aligned}$$

where

8 $$\begin{aligned} \left\langle \left[ f_{s,\Delta }^{(t)}(k)\right] ^2\right\rangle =W^{-1}\sum _{t=1}^{W}\left( f_{s,\Delta }^{(t)}(k)\right) ^2 \end{aligned}$$

It has been observed for router activity and email traffic (but also stock markets, river flows, or printing activity)^15^ that the standard deviation of the *i*-th entity’s activity (calculated in windows of size $\Delta$) scales with its mean with an exponent $\alpha$ (and a multiplicative constant *B*):

9 $$\begin{aligned} \tilde{\sigma }_{s,\Delta }(k) = B_\Delta \left\langle f_{s,\Delta }^{(t)}(k)\right\rangle ^{\alpha _\Delta }. \end{aligned}$$

Artificial examples of this procedure are shown in Fig. 1, explaining how a set of sources publishing about a single concept gives rise to TFS (middle column in Fig. 1) for different time windows.

Recent theoretical advances have greatly improved our understanding of mechanisms that underlie TFS in a given system^40,52–54^. One of the generative mechanisms is that entity activities in this class of systems can be described with Tweedie distributions; exponents between 1 and 2 suggests the compound Poisson-gamma distribution^55^. This model arises when in each time step activity of an entity is a sum of i.i.d. gamma-distributed random variables and the number of the random variables is drawn from a Poisson distribution.

In the case of news outlet activities, each random variable might be an occurrence of a real world event which causes a news outlet to write articles describing it; the number of articles per event follows a Gamma distribution. Each outlet can have a different threshold for an event importance or relevance (influencing the Poisson process rate) and/or attitude towards a topic (influencing parameters of the distribution of articles per event) which can account for heterogeneity in the number of articles and temporal activity fluctuations amount of the entities creating the system.

#### Agglomerative clustering of correlation matrices

To analyze relationships between residuals $R_{s,\Delta }(k)$ (see Eq. (1)) and Principal Components $P_{s,p}(k)$ (see the subsection below) for various combination of keywords *k* and time window sizes $\Delta$ or Principal Components *p*, we calculated correlation matrices. Each row/column of the matrix represents correlations between one pair $(k,\Delta )$ or one pair (*k*, *p*) with the remaining pairs. As keywords were reported by different numbers of publishers, it was necessary to calculate correlation coefficients using only publishers that covered both keywords. We used Pearson’s correlation coefficient; here for residuals, similarly for PCs:

10 $$\begin{aligned} \rho \left( k_1, \Delta _1; k_2, \Delta _2 \right) = {\frac{\sum \nolimits _{s\in S_{k_1,k_2}}\left( R_{s,\Delta _1}(k_1)-{\bar{R}_1}\right) \left( R_{s,\Delta _2}(k_2)-{\bar{R}_2}\right) }{{\sqrt{\sum \nolimits _{s\in \mathscr {S}_{k_1,k_2}}\left( R_{s,\Delta _1}(k_1) -{\bar{R}_1}\right) ^{2}}}{\sqrt{\sum \nolimits _{s\in \mathscr {S}_{k_1,k_2}}\left( R_{s,\Delta _2}(k_2) -{\bar{R}_2}\right) ^{2}}}}} \end{aligned}$$

where $R_{s,\Delta }(k)$—the residual of publisher *s* calculated for keyword *k* and time window size $\Delta$, $\mathscr {S}_{k}$—a set of publishers that published articles about a keyword *k*, $\mathscr {S}_{k_1,k_2} = \mathscr {S}_{k_1} \cap \mathscr {S}_{k_2}$, and $\bar{R}_x = \frac{1}{|\mathscr {S}_{k_1,k_2}|}\sum \nolimits _{s\in \mathscr {S}_{k_1,k_2}}R_{s,\Delta _x}(k_x)$.

To group combinations that were positively correlated, we performed Agglomerative Clustering (AC) of the matrices^56^. The usual inputs to clustering algorithms are dissimilarity (distance) matrices so we calculated distances between the $(k,\Delta )$ pairs as follows:

11 $$\begin{aligned} d\left( k_1, \Delta _1; k_2, \Delta _2 \right) =1-\rho \left( k_1, \Delta _1; k_2, \Delta _2 \right) \end{aligned}$$

AC is a bottom-up clustering method—the algorithm is initialized with each $(k,\Delta )$ combination in a separate cluster. Then, at each step, the nearest pair of clusters is merged. We selected the unweighted pair group method with arithmetic mean (UPGMA) which defines the distance between clusters *A* and *B* as the arithmetic mean of the distances between objects in the clusters:

12 $$\begin{aligned} d(\mathscr {A},\mathscr {B}) = \frac{1}{|\mathscr {A}||\mathscr {B}|}\sum \limits _{a\in \mathscr {A}} \sum \limits _{b\in \mathscr {B}}d(a,b). \end{aligned}$$

The result of the clustering can be drawn as a rooted tree (a dendrogram) and used to sort the rows/columns of a correlation matrix.

#### Principal component analysis (PCA)

Despite being over a hundred years old^57^, PCA is commonly used in contemporary exploratory analyses of highly dimensional datasets^25,58–60^. This method transforms an original set of variables to so-called Principal Components (PC). PCs are mutually orthogonal linear combinations of the original variables. The first PC is a linear combination of the original variables so that the variance of the data along the axis is maximized; the remaining PCs are constructed in a similar way with additional requirements of orthogonality to all the previous PCs. The main PCA applications are visualization and dimensionality reduction. Nowadays, the procedure is usually carried out using Singular Value Decomposition (SVD) of the data matrix (or, equivalently, Eigenvalue Decomposition of the data covariance matrix). The *scikit-learn*, a Python machine learning library^61^ which we performed all the PCA procedures in the study with, uses the LAPACK implementation of the SVD^62^.

**Figure 8:**
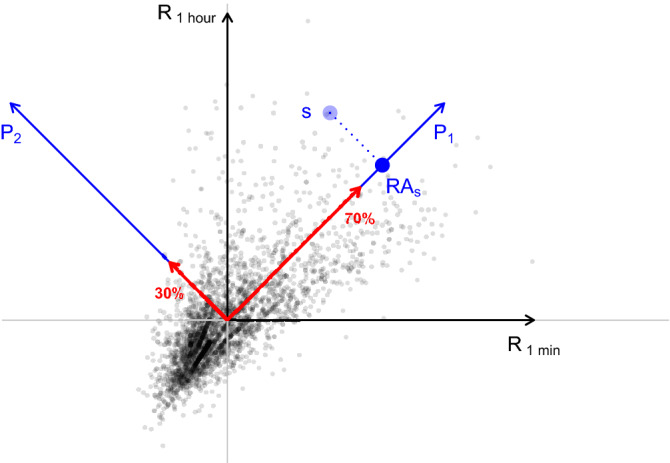
Illustration of PCA dimension reduction. Points are original data—residuals coming from the *Terrorism* keyword for two selected time windows: 1 min ($R_{1{\text { min}}}$) and 1 h ($R_{1{\text { h}}}$). Blue vectors show directions of the new set of variables obtained by the PCA method: first ($P_1$) and second ($P_2$) Principal Components. While red arrows mark the amount of variance explained by these variables (the length of each arrow is proportional to the variance explained by the respective PC). The blue point is a selected source *s* and the blue dotted line shows the projection of this data point onto the first PC which is this source’s reactivity $RA_s$.

In the paper, we denote the decomposition of a matrix $\hat{\mathbf {R}}$ that includes the original data vectors into PCs as follows:

13 $$\begin{aligned} \hat{\mathbf {R}} = \hat{\mathbf {P}}\hat{{\mathbf {G}}}^T \iff \hat{\mathbf {P}} = \hat{\mathbf {R}}\hat{{\mathbf {G}}} \end{aligned}$$

where a matrix $\hat{\mathbf {P}}$ contains projections of the original variables to principle components, and $\hat{{\mathbf {G}}}$ is a matrix of new basis vectors: a transformation matrix for old variables to principle components.

One of the most important features of PCA is its ability to detect linear correlations among the components of data vectors and to indicate the amount of variance explained by each of the newly-introduced variables, i.e., PCs. If the consecutive PCs explain a considerable amount of variance, e.g., 90%, the remaining variables can be ignored. This is known as dimensionality reduction. In the case of our data we make use of the first PC which usually explains over 60% of variance (cf Fig. 4).

As it is impossible to visualize 14 dimensions, Fig. 8 shows residuals for two selected dimensions—1 min ($R_{1{\text { min}}}$) and 1 h ($R_{1{\text { h}}}$) for the keyword *Terrorism*. The plot shows that $R_{1{\text { min}}}$ and $R_{1{\text { h}}}$ are not independent: the larger $R_{1{\text { min}}}$ the larger $R_{1{\text { h}}}$, so they should not be treated as a valid set of coordinates. As an outcome of PCA we obtain two new directions—$P_1$ and $P_2$ that explain, respectively, 70% and 30% of the total variance and create a better setting to describe this data. The value of $P_1$, i.e., the projection of the original variables onto the first PC, is the *reactivity*, examined in this study.

#### Kruskal–Wallis test and Dunn’s post-hoc test

To check for differences in relative median reactivity $\tilde{B}_b(k)$ between bias groups, we performed Kruskal–Wallis tests^63^. After a significant Kruskal–Wallis test, pairwise comparisons were performed to order bias groups by increasing $\mathrm {median}\,\tilde{B}_b$. The *p*-values were FDR adjusted using the two-step Benjamini–Krieger–Yekutieli method^32^. In our study, we used the *scikit-posthocs*^64^ implementation of these tests.

## Supplementary information

Supplementary Information.

## Data Availability

The dataset analyzed in the study (lists of news publication timestamps by keyword and publisher) is available from the corresponding author on reasonable request.

## Code availability

Python scripts for calculating TFS and reactivity from a list of timestamps as well as scripts that aggregate reactivity are available from the corresponding author on reasonable request.
