# Supplementary material for: A calibrated measure to compare fluctuations of different entities across timescales
Source: Sci Rep. 2020 Nov 26;10:20673. doi: 10.1038/s41598-020-77660-4 (PMC7691371; doi:10.1038/s41598-020-77660-4)
Supplement: Supplementary file 1 — Supplementary Information. [file 41598_2020_77660_MOESM1_ESM.pdf]

A calibrated measure to compare fluctuations of different entities across timescales
SUPPLEMENTARY INFORMATION

Jan Chołoniewski[1], Julian Sienkiewicz[1], Naum Dretnik[2], Gregor Leban[3], Mike Thelwall[4], and Janusz A. Hołyst[1,5,*]

[1]Center of Excellence for Complex Systems Research, Faculty of Physics, Warsaw University of Technology, Koszykowa 75, 00-662, Warsaw, Poland
[2]Slovenian Press Agency, Tivolska 48, 1000, Ljubljana, Slovenia
[3]Artificial Intelligence Laboratory, Jožef Stefan Institute, Jamova 39, 1000, Ljubljana, Slovenia
[4]School of Mathematics and Computing, University of Wolverhampton, Wulfruna Street, Wolverhampton WV1 1LY, UK
[5]ITMO University, 49 Kronverkskiy av., 197101, Saint Petersburg, Russia
*janusz.holyst@pw.edu.pl

Contents

1 Additional dataset info 1

2 Dataset cleaning 1

2.1 Types of erroneous timeseries 1

2.2 Toy models for erroneous timeseries 3

2.3 Applying the models to the clean dataset 5

3 Temporal Fluctuation Scaling in the Event Registry dataset 7

4 Aggregated correlation matrices 9

5 Aggregated reactivity 14

6 Measure comparisons 18

1 Additional dataset info

Example timeseries can be found in Fig. S1. Numbers of articles and sources as well as values of the TFS scaling exponent for selected time window sizes can be found in the Tab. S1.

2 Dataset cleaning

2.1 Types of erroneous timeseries

For effective aggregation of TFS residuals, it was necessary check for outliers. We identified two types of outliers corresponding to two common ER crawler malfunctions:

1. unusually many of a given publisher's articles annotated with a given concept in a very short period (typically from a few minutes to a few hours). This is typically due to changes in website layouts that confuse a crawler in one of two ways: causing it to misidentify a batch of old articles as new, adding them with a new date or corrupting text extraction to include parts of other articles or unrelated texts, causing incorrect semantic annotations.

   Timeseries corrupted in this way have abnormally increased $R(\Delta)$ for each Δ as there is only a slight dependence on Δ in Eq.1. The most significant terms are constant and $\gg 1$.

concept	# articles	# sources	with known bias	$\alpha(\Delta = 1\,\text{hour})$	$\alpha(\Delta = 1\,\text{day})$	$\alpha(\Delta = 30\,\text{days})$
United States	4,822,745	6,299	584	0.542(0.001)	0.624(0.002)	0.793(0.005)
United Kingdom	2,790,790	4,997	514	0.538(0.001)	0.619(0.002)	0.799(0.005)
France	2,445,135	4,255	415	0.536(0.001)	0.621(0.002)	0.817(0.005)
Germany	2,209,710	4,521	434	0.536(0.001)	0.614(0.002)	0.804(0.005)
China	1,966,620	4,316	497	0.537(0.001)	0.612(0.002)	0.772(0.006)
European Union	1,782,295	3,316	381	0.538(0.001)	0.630(0.002)	0.816(0.006)
Facebook	1,675,350	4,615	513	0.528(0.001)	0.602(0.002)	0.776(0.006)
Japan	1,226,765	3,861	418	0.534(0.001)	0.603(0.002)	0.776(0.007)
Terrorism	1,197,565	2,987	424	0.530(0.001)	0.607(0.003)	0.764(0.007)
India	1,179,680	3,088	370	0.547(0.001)	0.621(0.003)	0.811(0.008)
Brazil	1,121,280	3,072	316	0.536(0.001)	0.628(0.003)	0.836(0.007)
Mexico	1,022,000	3,210	452	0.537(0.001)	0.637(0.003)	0.837(0.007)
Argentina	965,060	2,608	242	0.547(0.001)	0.618(0.003)	0.799(0.007)
Iran	881,475	2,399	363	0.533(0.001)	0.633(0.003)	0.788(0.007)
Shooting	669,410	2,041	472	0.530(0.001)	0.624(0.003)	0.827(0.008)
Egypt	631,815	2,046	281	0.542(0.001)	0.630(0.004)	0.827(0.010)
Real Madrid C.F.	581,080	1,544	107	0.534(0.001)	0.604(0.004)	0.743(0.009)
Austria	579,255	2,043	194	0.528(0.001)	0.597(0.003)	0.761(0.009)
Indonesia	549,325	1,701	217	0.543(0.001)	0.640(0.004)	0.824(0.009)
Poland	522,680	2,201	229	0.524(0.001)	0.591(0.003)	0.750(0.009)
North Korea	519,030	1,934	349	0.531(0.001)	0.661(0.003)	0.824(0.007)
South Africa	498,955	2,008	260	0.540(0.001)	0.619(0.003)	0.818(0.010)
Islam	477,420	1,868	273	0.529(0.001)	0.590(0.003)	0.748(0.010)
Cannabis (drug)	303,680	1,842	351	0.528(0.001)	0.607(0.004)	0.789(0.012)
Morocco	301,125	1,490	144	0.539(0.001)	0.631(0.005)	0.824(0.012)
Climate change	271,560	1,680	347	0.524(0.001)	0.578(0.004)	0.733(0.013)
Universe	249,295	1,671	305	0.524(0.001)	0.572(0.003)	0.714(0.013)
Capital punishment	176,660	1,224	248	0.526(0.001)	0.621(0.005)	0.786(0.014)
Slovenia	157,680	858	69	0.529(0.002)	0.577(0.005)	0.752(0.015)
Abortion	138,335	1,047	266	0.529(0.002)	0.626(0.006)	0.810(0.015)
Bitcoin	127,020	676	149	0.525(0.003)	0.631(0.007)	0.865(0.015)
Homosexuality	113,515	1,018	208	0.519(0.002)	0.569(0.005)	0.689(0.018)
Roger Federer	75,190	546	73	0.528(0.002)	0.608(0.008)	0.748(0.019)
Marvel Comics	64,605	436	122	0.520(0.002)	0.569(0.007)	0.758(0.021)
Same-sex marriage	47,450	460	163	0.527(0.003)	0.628(0.010)	0.805(0.026)
Kim Kardashian	45,260	377	83	0.515(0.002)	0.562(0.009)	0.739(0.027)
British royal family	40,880	328	83	0.541(0.004)	0.637(0.014)	0.817(0.025)
Blockchain	16,790	100	23	0.559(0.010)	0.798(0.013)	0.995(0.015)
Usain Bolt	6,205	88	22	0.541(0.012)	0.641(0.033)	0.951(0.077)

Table S1. Concepts considered in the study with basic articles and publishers statistics and TFS exponents for $\Delta \in \{1\,\text{hour}, 1\,\text{day}, 30\,\text{days}\}$. Only sources that published at least three articles per month about a given concept. Articles from other sources were discarded.

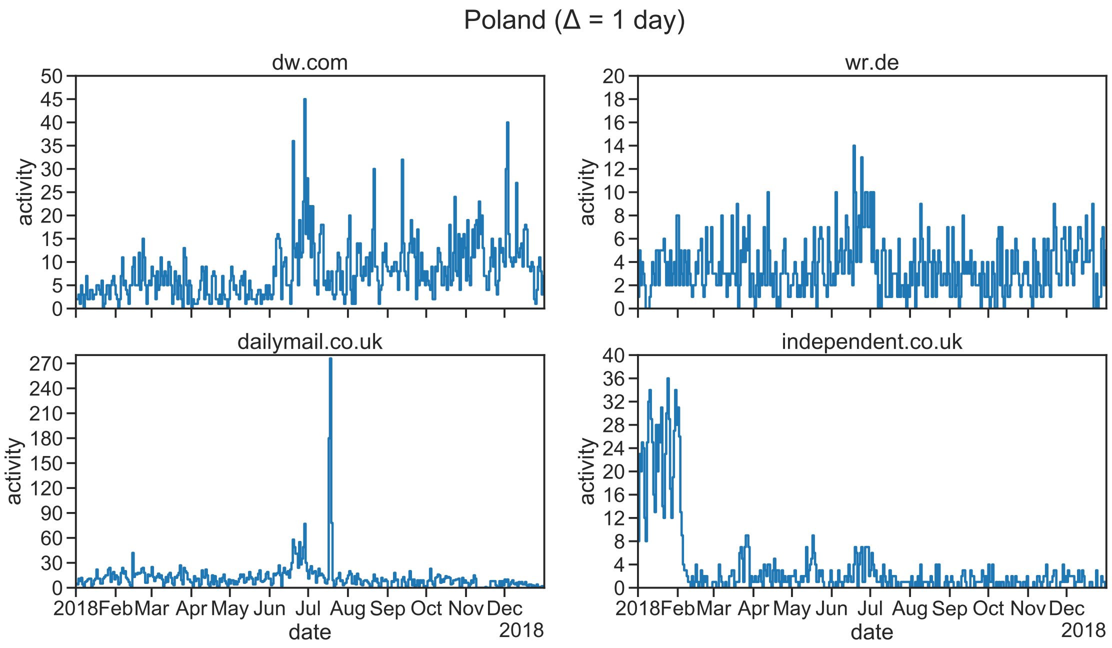

Figure S1. Example activity timeseries for keyword *Poland* with $\Delta = 1$ day resolution. Examples of (top-left) high reactivity (raw data: $PC1 = 1.43$, $PC2 = 1.33$, clean data: $PC1 = 6.1$, $PC2 = 0.17$), (top-right) low reactivity (raw data: $PC1 = -2.31$, $PC2 = -0.95$, clean data: $PC1 = -1.52$, $PC2 = -2.13$), (bottom-left) error type 1 (raw data: $PC1 = 11.42$, $PC2 = -0.51$), (bottom-right) error type 2 (raw data: $PC1 = 7.22$, $PC2 = 4.5$).

2. Missing data in some period; the most common reason is the lack of functional crawler (not created yet or because of a layout change).

   Timeseries corrupted in this way have abnormally increased $R(\Delta)$ for higher Δ (because then $\alpha > 0.5$).

Examples of timeseries with type 1 and type 2 errors can be found in Fig. S1. Detailed theoretical and numerical consideration of these two models can be found below.

2.2 Toy models for erroneous timeseries

In the main paper, we defined a TFS residual as:

$$R_s^k(\Delta) = \log_{10} \frac{y_s(\Delta)}{10^{\beta_k(\Delta)} x_s(\Delta)^{\alpha_k(\Delta)}} = \log_{10} y_s(\Delta) - \alpha_k(\Delta) \log_{10} x_s(\Delta) - \beta_k(\Delta) \tag{1}$$

Let $f^{(q,\Delta)}$ be a timeseries with a mean μ and a variance σ^2. We will now use it to construct toy models of the two mentioned corrupted timeseries and check their influence on the residual values:

1. In the first scenario a large number of articles is added in a short period. Assuming that the period is shorter than the shortest windows Δ, we propose a toy model where one element is increased by some large value $g^{(q=q_1,\Delta)} = f^{(q=q_1,\Delta)} + V$ and other elements are unchanged: $g^{(q\neq q_1,\Delta)} = f^{(q,\Delta)}$.

   This causes the mean value to change (introducing $\delta = \frac{\Delta}{T}$ – a ratio of a time window size to the total observed period, and simplifying the notation with $f = f^{(q,\Delta)}$ and $g = g^{(q,\Delta)}$):

$$\langle g \rangle = \langle f \rangle + \delta V = \langle f \rangle \left(1 + \delta \times \frac{V}{\langle f \rangle} \right) \tag{2}$$

   Similarly:

$$\langle g^2 \rangle = \langle f^2 \rangle + \frac{\Delta}{T} \left(V^2 + 2V f^{(q_1,\Delta)} \right) \tag{3}$$

Thus the variance:

$$(\sigma_g^\Delta)^2 = \left\langle g^2 \right\rangle - \langle g \rangle^2 = (\sigma_f^\Delta)^2 + \delta\left(V^2 + 2V f^{(q_1,\Delta)}\right) - (\delta V)^2 - 2\delta V \langle f \rangle \tag{4}$$

Knowing that $V > 0$:

$$(\sigma_g^\Delta)^2 = (\sigma_f^\Delta)^2 + \delta\left(1 + 2\frac{f^{(q_1,\Delta)} - \langle f \rangle}{V} - \frac{\Delta}{T}\right) V^2 \approx (\sigma_f^\Delta)^2 + \delta(1-\delta) V^2 \tag{5}$$

$$(\sigma_g^\Delta)^2 \approx (\sigma_f^\Delta)^2 \left(1 + \delta(1-\delta)\frac{V^2}{(\sigma_f^\Delta)^2}\right) \tag{6}$$

Putting Eqs.2 and 6 to Eq. 1, we have:

$$R_g(\Delta) = \log_{10}\sigma_g^\Delta - \alpha(\Delta)\log_{10}\langle g \rangle - \beta(\Delta) = \tag{7}$$

$$= R_f(\Delta) + \frac{1}{2}\log_{10}\left(1 + \delta(1-\delta)\frac{V^2}{(\sigma_f^\Delta)^2}\right) - \alpha(\Delta)\log_{10}\left(1 + \delta \times \frac{V}{\langle f \rangle}\right) \tag{8}$$

Timeseries corrupted in this way have abnormally increased $R(\Delta)$ for each Δ as there is only a slight dependence on Δ in the last equation. The most significant terms are constant and $>> 1$.

2. In the second scenario, for a period of time no articles are added. A toy model for this case would be a timeseries $f^{(q,\Delta)}$ with a fraction p of values is replaced with zeros: $h^{(q<q_2,\Delta)} = 0$ where $q_2 = \lfloor pT \rfloor$. Then the mean (introducing $h = h^{(q,\Delta)}$):

$$\langle h \rangle = (1-p)\langle f \rangle \tag{9}$$

Assuming the squared values of f for $q \le q_2$ and $q > q_2$ are equally distributed:

$$\left\langle h^2 \right\rangle = (1-p)\left\langle f^2 \right\rangle \tag{10}$$

Hence the variance:

$$(\sigma_h^\Delta)^2 = (1-p)\left\langle f^2 \right\rangle - (1-p)^2\langle f \rangle^2 = (1-p)(\sigma_f^\Delta)^2 + p(1-p)\langle f \rangle^2 = \tag{11}$$

$$= (1-p)(\sigma_f^\Delta)^2\left(1 + p\frac{\langle f \rangle^2}{(\sigma_f^\Delta)^2}\right) \tag{12}$$

Putting Eqs. 9 and 12 to Eq. 1, we have:

$$R_g(\Delta) = \log_{10}\sigma_h^\Delta - \alpha(\Delta)\log_{10}\langle h \rangle - \beta(\Delta) = \tag{13}$$

$$= R_f(\Delta) + \left(\frac{1}{2} - \alpha(\Delta)\right)\log_{10}(1-p) + \frac{1}{2}\log_{10}\left(1 + p\frac{\langle f \rangle^2}{(\sigma_f^\Delta)^2}\right) \tag{14}$$

Timeseries corrupted in this way have abnormally increased $R(\Delta)$ for higher Δ (because then $\alpha > 0.5$).

We checked the correctness of our toy models by modifying original timeseries with one of the two disturbances (activity spike or periods of missing data) and comparing it to results predicted by our calculations. Visualizations of both models with different parameters – Fig. S2. For the spike model, the results are almost the same as predicted. For the missing data model, there are minor deviations from the theory caused by a violation of the assumption of equal activity distributions in the whole timeseries.

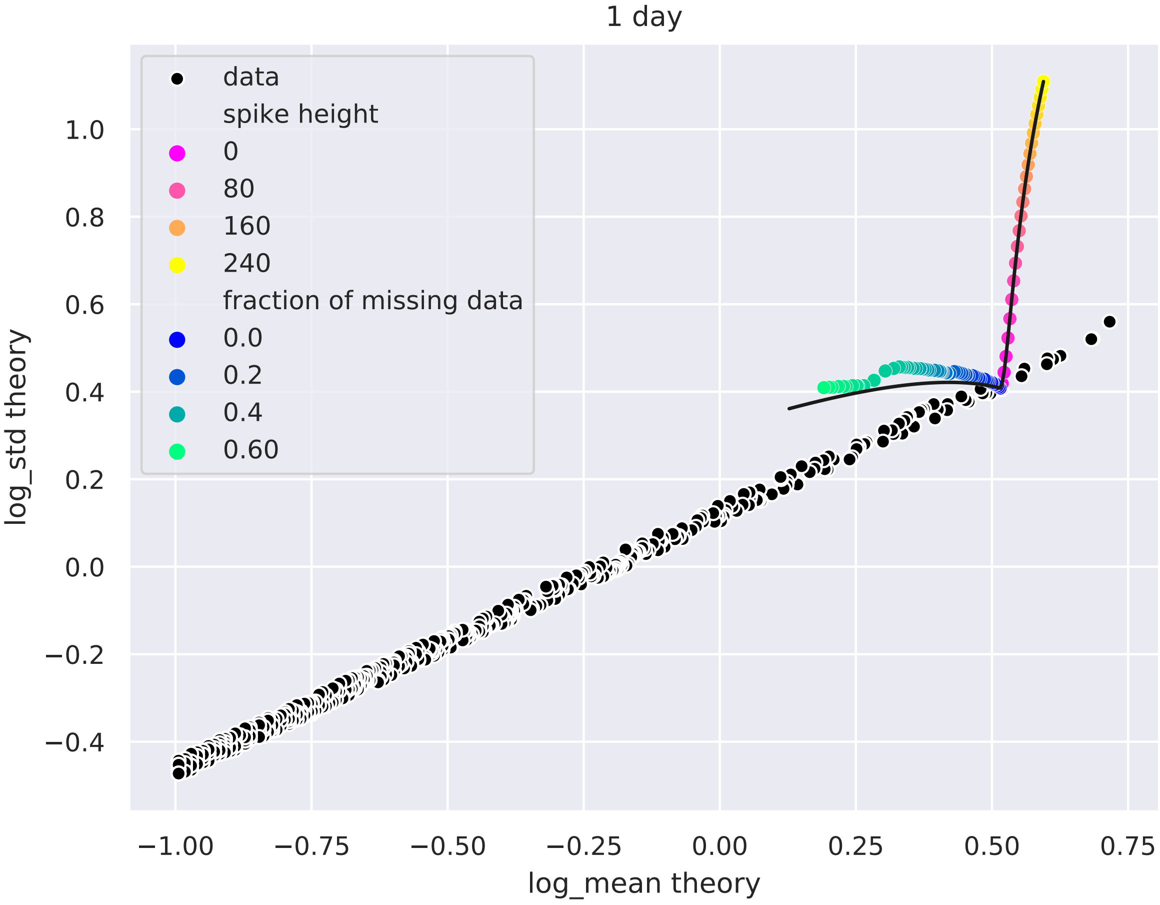

Figure S2. Effects of two types of disturbances (activity spike, periods of missing data) on a source position in TFS plots. Black points are original (filtered) data, colored points are activity timeseries modified with one of the two models with various parameters, black curves are theoretical fits for the same range of parameters values.

2.3 Applying the models to the clean dataset

As a result of the discussion above, it is clear that considering corrections in only one timescale might be insufficient as the correction can vary for different time windows. To properly identify sources with corrupted timeseries, we performed Principal Component Analysis of a matrix of corrections calculated for all sources. For a keyword k, we constructed a matrix M^k with rows representing news outlets s and columns standing for corrections for each Δ. In Fig. S3 there are visualizations of contributions of corrections for each Δ to first four principal components (matrix Π), and a cumulative explained variance ratio by PCs. The results are very similar in all cases. The first four PCs explain more than 95% of the variance. The first PC has approximately equal contributions from correction in all the analyzed time window sizes; in the second PC, contributions from the short time window corrections have an opposite sign to contributions from the long time windows. As the experimental composition of the two PCs is coincident to the effects described above, we interpret sources with a high absolute PC1 value as type-1 corrupted, and sources with PC2 values associated with high positive values of corrections in longer time windows as type-2 corrupted (sources with oppositely signed PC2 values tend to add many articles in very short time periods which we suspect to reflect rather a publishing strategy than an effect of crawler malfunction). However, erroneous timeseries cannot be perfectly separated from correct timeseries using the method and so the filtering needs to be performed using threshold values.

Exact identification of timeseries with errors was time-consuming as it often required analyzing positions of unexpected peaks and/or browsing articles in a suspected period to evaluate whether a change in the timeseries character was a result of the errors described or an unconventional publisher's behavior. Thus we identified publishers with corrupted timeseries by an inspection of randomly sampled timeseries with gradually less extreme values of PC1 and/or PC2. Experimentally, we

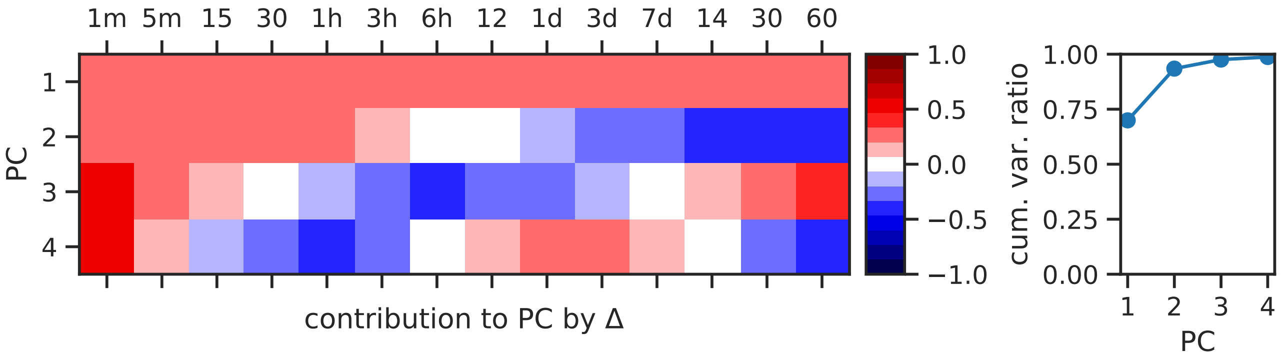

Figure S3. Principal Component Analysis of residuals by time window size for keyword *European Union* (raw data). (left) Contributions of corrections for different Δ to first four PCs (matrix Π). (right) A cumulative explained variance ratio for first four PCs.

found that a threshold value 2.5 (PC1 for type 1 errors, PC2 for type 2 errors) was guaranteeing satisfying precision and recall trade-off. We discarded publishers with PC1 or PC2 above this value and rerun the calculations of TFS exponents and residuals to account for changes in results caused by the filtering procedure.

3 Temporal Fluctuation Scaling in the Event Registry dataset

We have reported the fit of the TFS for online news outlet activities in our previous paper. While outliers have only a slight impact for the value of the TFS exponent, here we report results which heavily rely on the correctness of the dataset. Because of this we developed a method to discard news outlets with corrupted activity timeseries – see above. In the Fig. S4, there is an example TFS plot for news outlet publishing around the topic *China* for the raw data (top-left) and for the cleaned data (top-right) with a time window size $\Delta = 1\,\text{day}$. The number of publishers with abnormally high activity variance is significantly reduced for the cleaned data. Moreover, two plots in the bottom panels of Fig. S4 show TFS residuals for raw (bottom-left) and cleaned (bottom-right) datasets. Comparing figures in the two top panels shows that outliers tend to drift towards higher fluctuations, as expected. Comparing the two bottom plots suggests that removing the outliers not only improves the linear fit ($R^2 \approx 0.85$ for raw data; $R^2 \approx 0.95$ for cleaned data) but also causes the cloud of points to be symmetrical with respect to the OX axis. TFS exponents for each concept for three selected time window sizes ($\Delta \in \{1\,\text{hour}, 1\,\text{day}, 30\,\text{days}\}$) can be found in Tab. S1. The dependence of the TFS exponent α_k on a time window size Δ is the same as in the previous study and can be found in Figs. S4, S5, and S6.

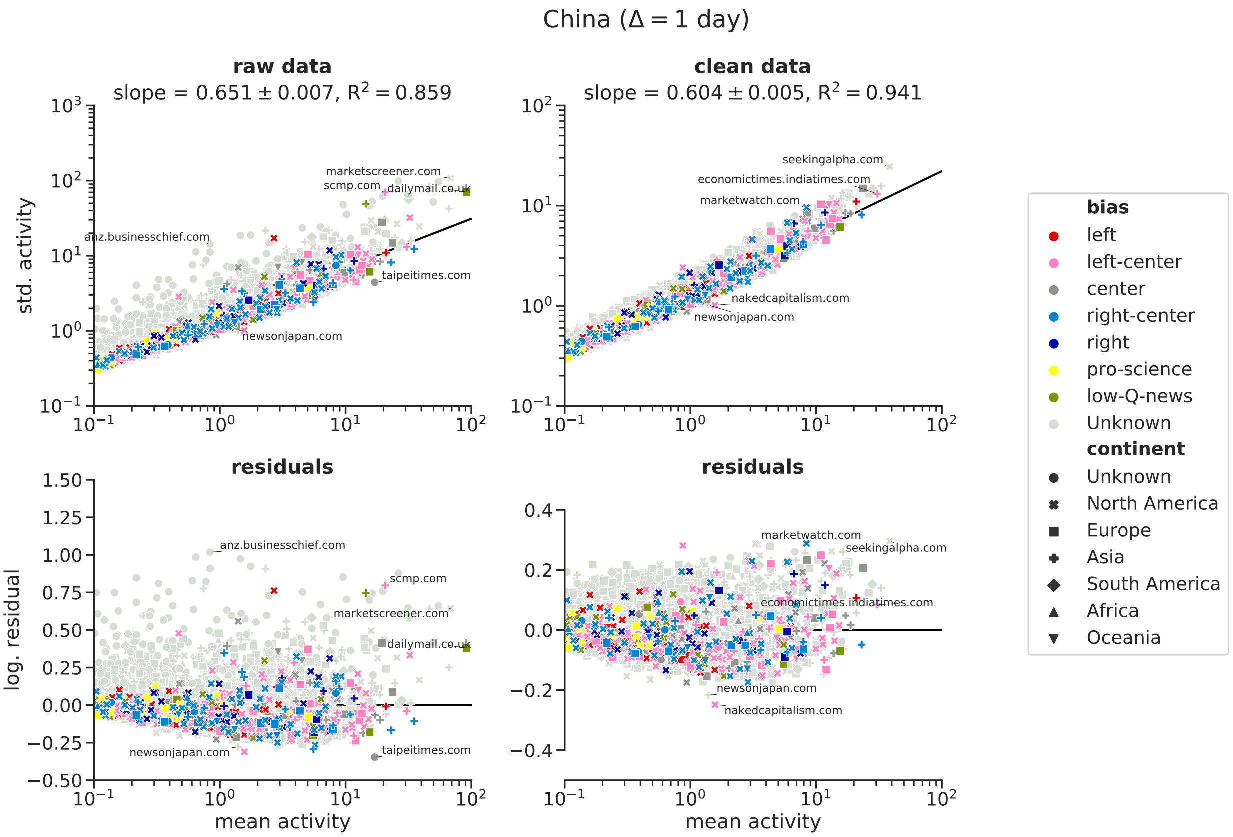

Figure S4. Fluctuation scaling and residuals for a concept *China* and a time window size $\Delta = 1,$day. (Left) raw data, (right) cleaned data, (top) fluctuation scaling, (bottom) TFS residuals.

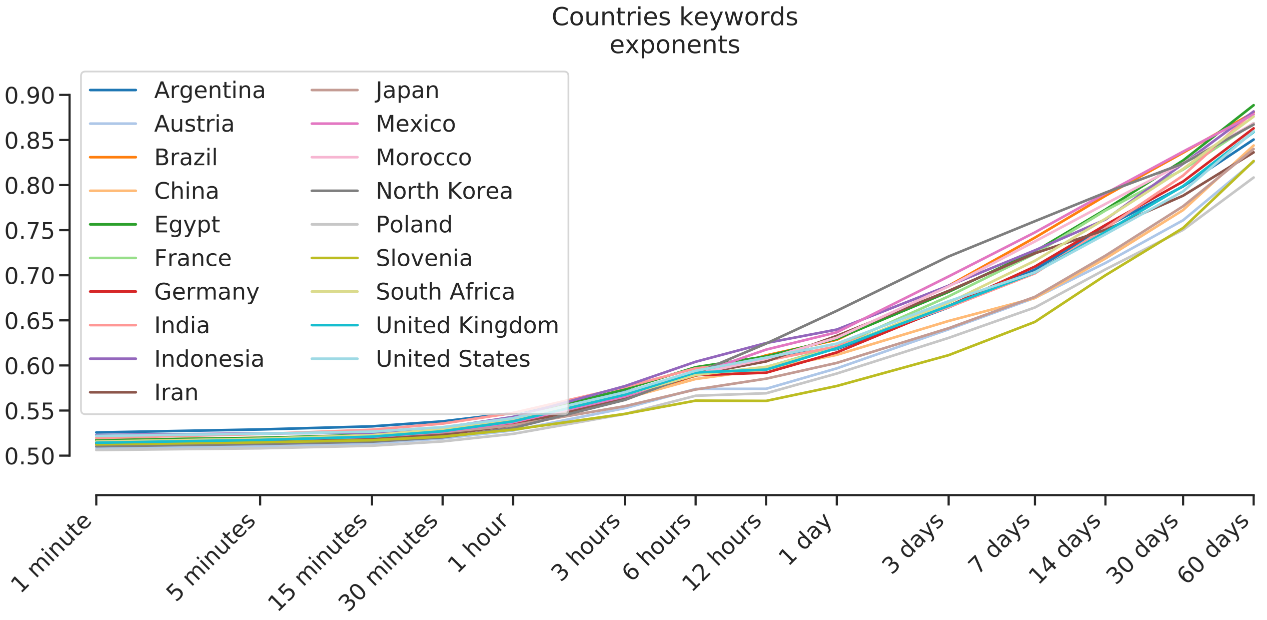

Figure S5. Dependence of the TFS exponent α on time window size Δ for keywords related to countries. X-axis is Δ, Y-axis is α.

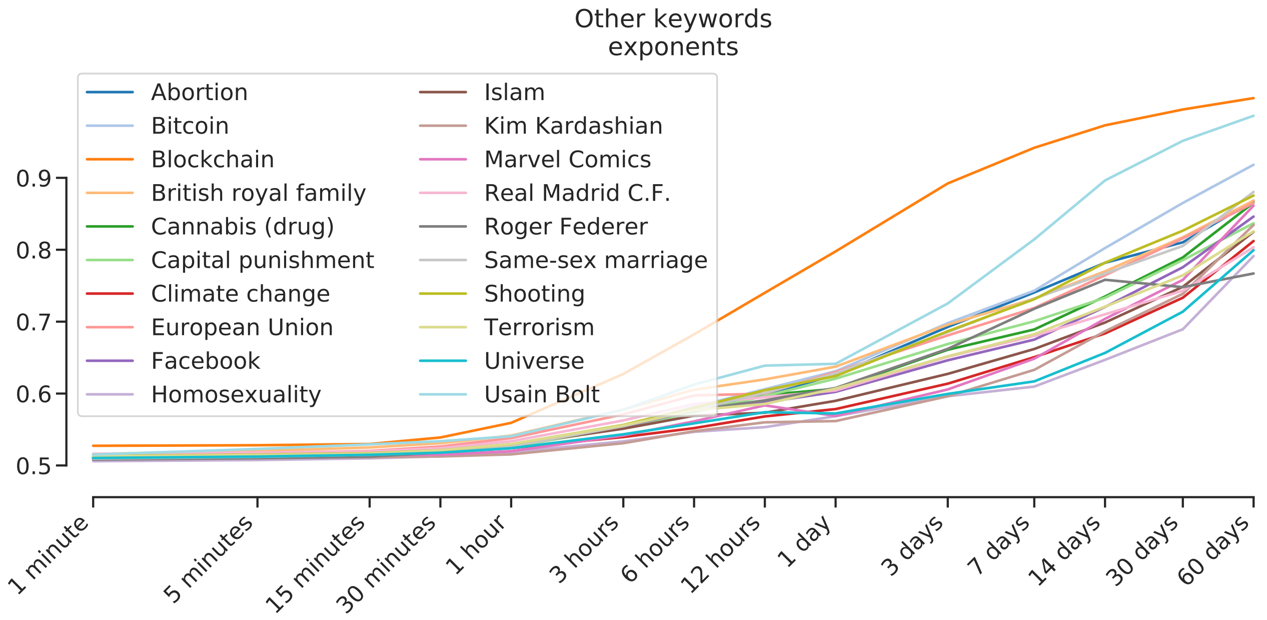

Figure S6. Dependence of the TFS exponent α on time window size Δ for other keywords (not related to countries). X-axis is Δ, Y-axis is α.

4 Aggregated correlation matrices

Matrices from Fig. 3 and 5 from the main text were averaged over keywords and time window sizes to observe trends. When aggregating by concept groups, for a given pair (k_1, k_2), a value in the averaged matrix $\hat{\mathbf{K}}$ was calculated as follows:

$$K(k_1, k_2) = \frac{1}{D(D-1)} \sum_{\Delta \in \mathscr{D}} \sum_{\Delta' \neq \Delta} \rho(k_1, \Delta; k_2, \Delta') \tag{15}$$

When aggregating in time window size groups, for a pair (Δ_1, Δ_2), a value in the averaged matrix $\hat{\mathbf{T}}$ was calculated as follows:

$$T(\Delta_1, \Delta_2) = \frac{1}{K(K-1)} \sum_{k \in \mathscr{K}} \sum_{k' \neq k} \rho(k, \Delta_1; k', \Delta_2) \tag{16}$$

For the window size groups ($\mathscr{D}_{short/medium/long}$), the values were calculated as follows:

$$T(\mathscr{D}_1, \mathscr{D}_2) = \frac{1}{|\mathscr{D}_1||\mathscr{D}_2|K(K-1)} \sum_{\Delta_1 \in \mathscr{D}_1} \sum_{\Delta_2 \in \mathscr{D}_2} \sum_{k \in \mathscr{K}} \sum_{k' \neq k} \rho(k, \Delta_1; k', \Delta_2) \tag{17}$$

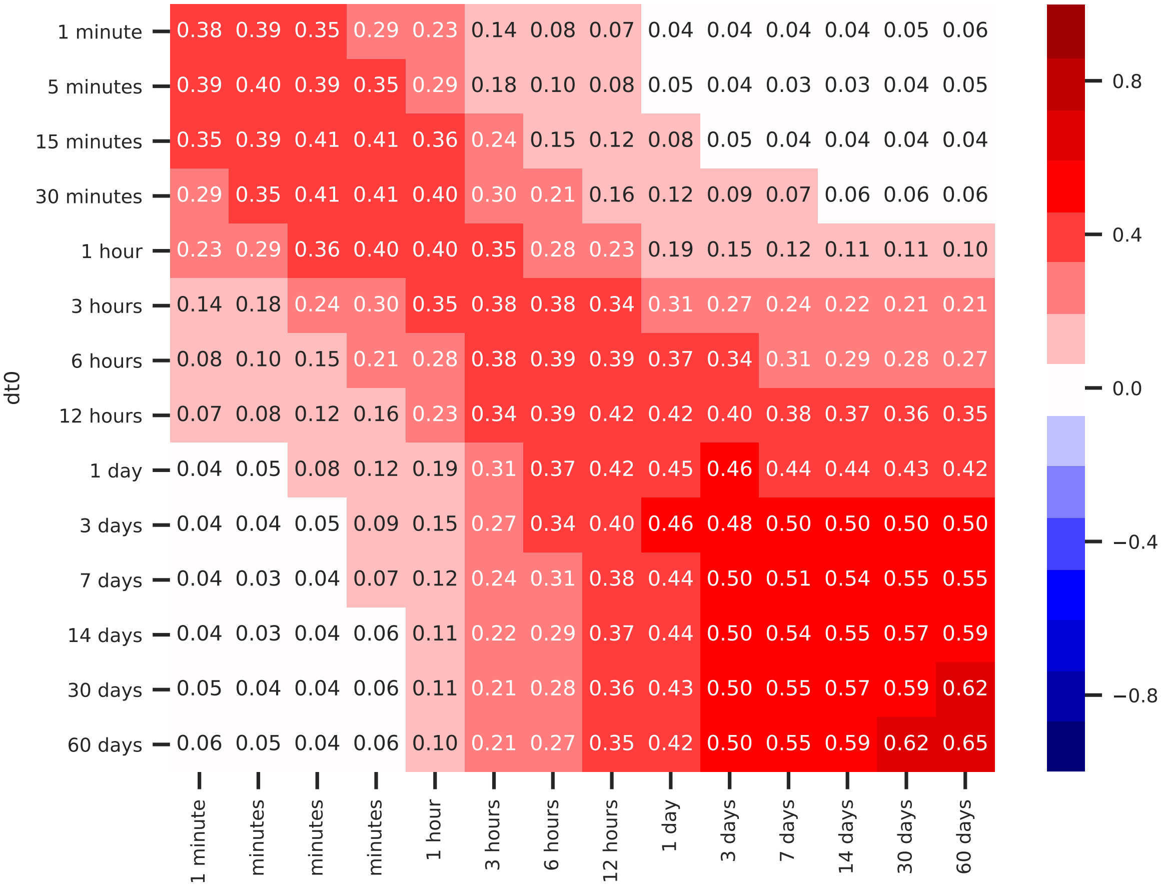

Figure S7. The correlation matrix of residuals (Fig. 3 in the main text) averaged over concepts in time window size groups. See Eq. 16 for the averaging procedure details.

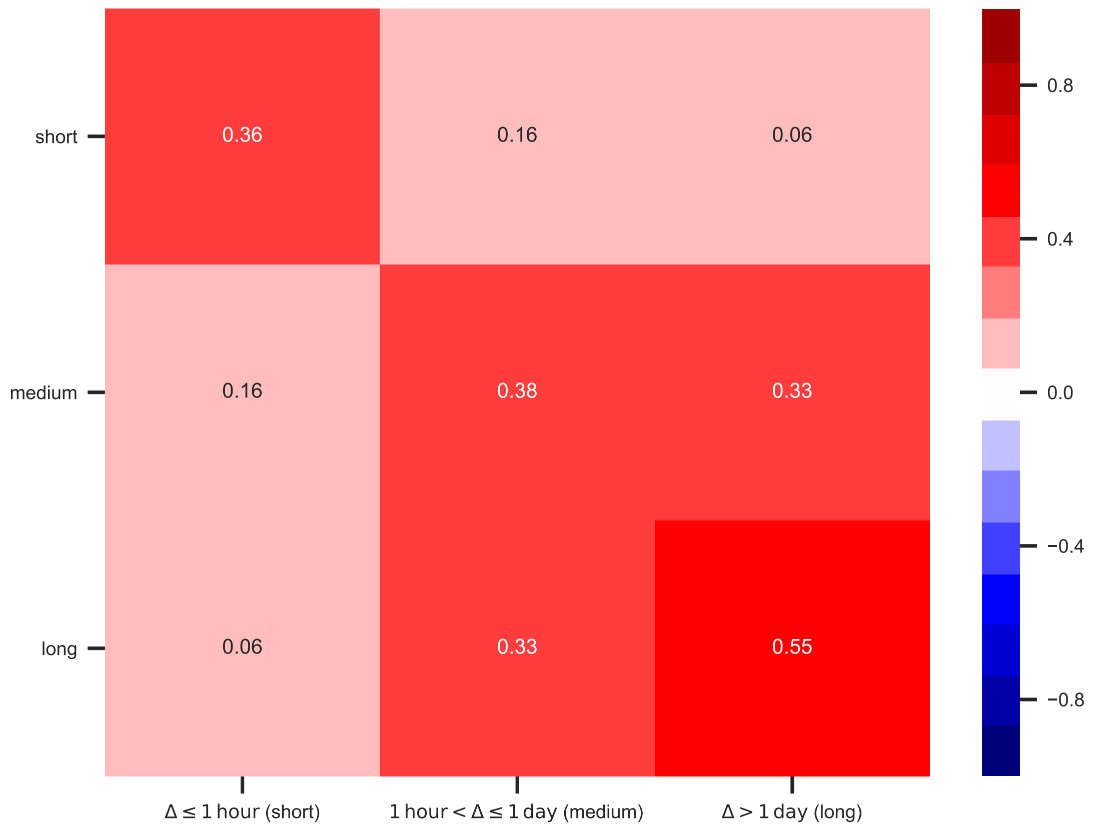

Figure S8. The correlation matrix of residuals (Fig. 3 in the main text) averaged over concepts in time window size groups. The averaging procedure was performed as in Eq. 17.

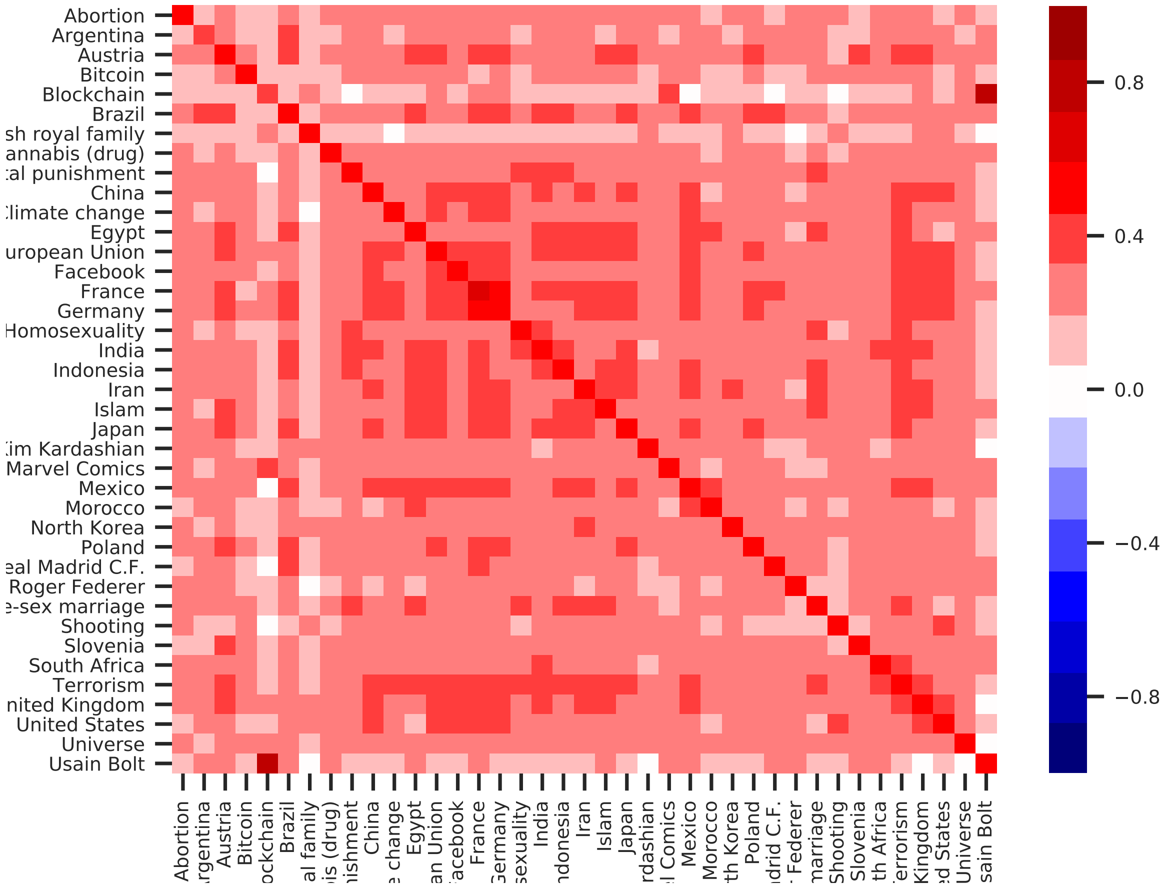

Figure S9. **The correlation matrix of residuals (Fig. 3 in the main text) averaged over time window sizes in groups by keyword.** See Eq. 15 for the averaging procedure details.

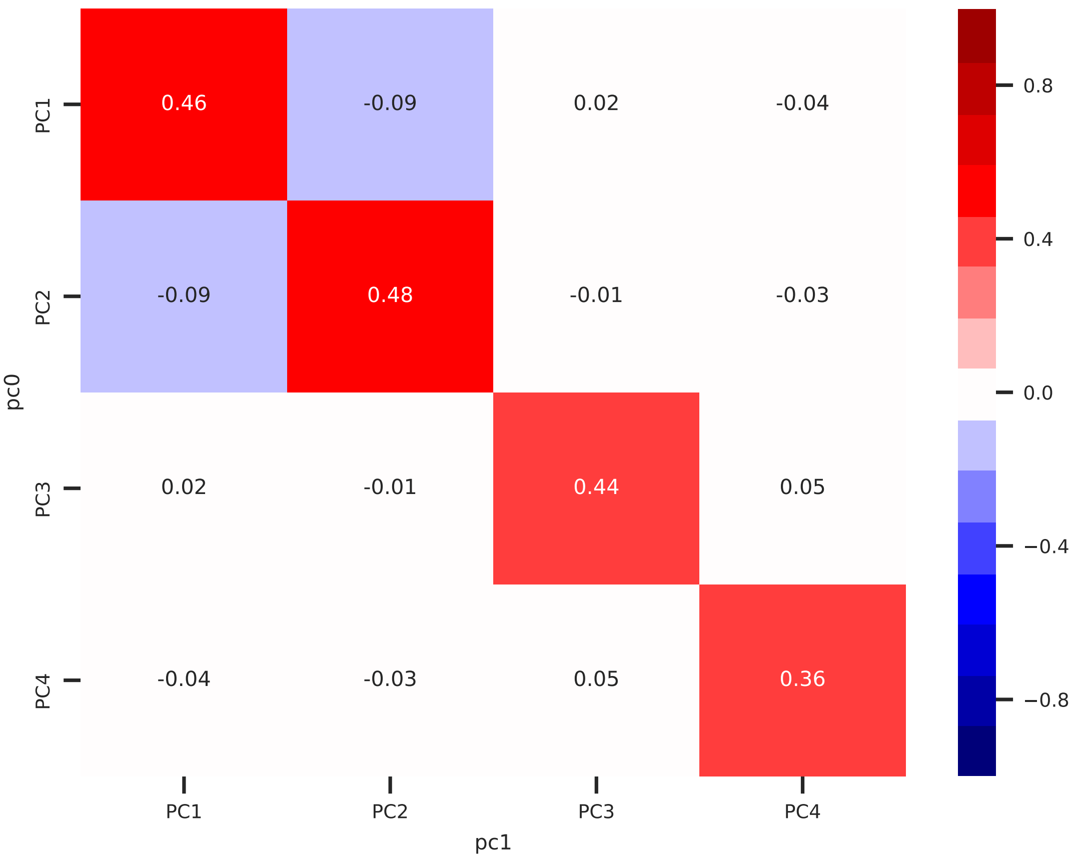

Figure S10. **The correlation matrix of PCs (Fig. 5 in the main text) averaged over keywords in groups by PC order.** The averaging procedure was performed as in Eq. 16.

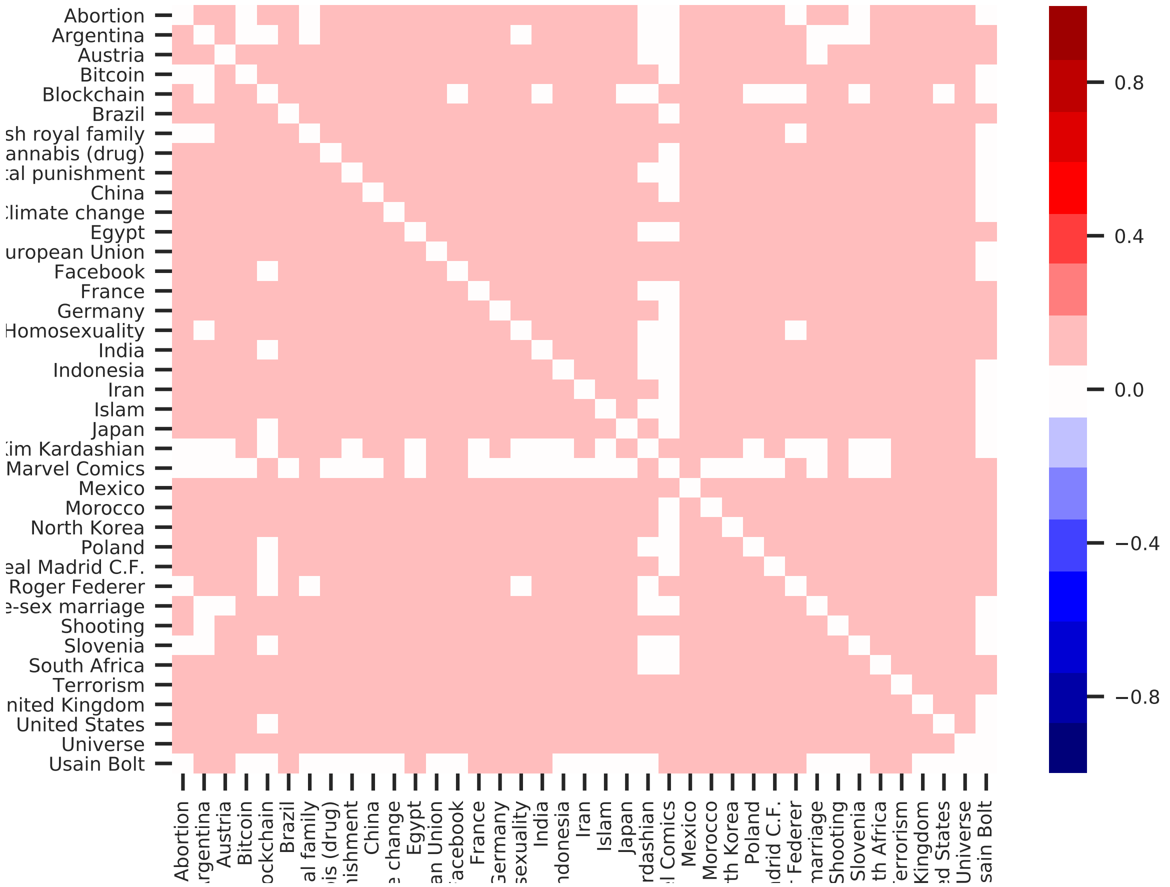

Figure S11. **The correlation matrix of PCs (Fig. 5 in the main text) averaged over PCs in groups by keyword.** See Eq. 15 for the averaging procedure details.

5 Aggregated reactivity

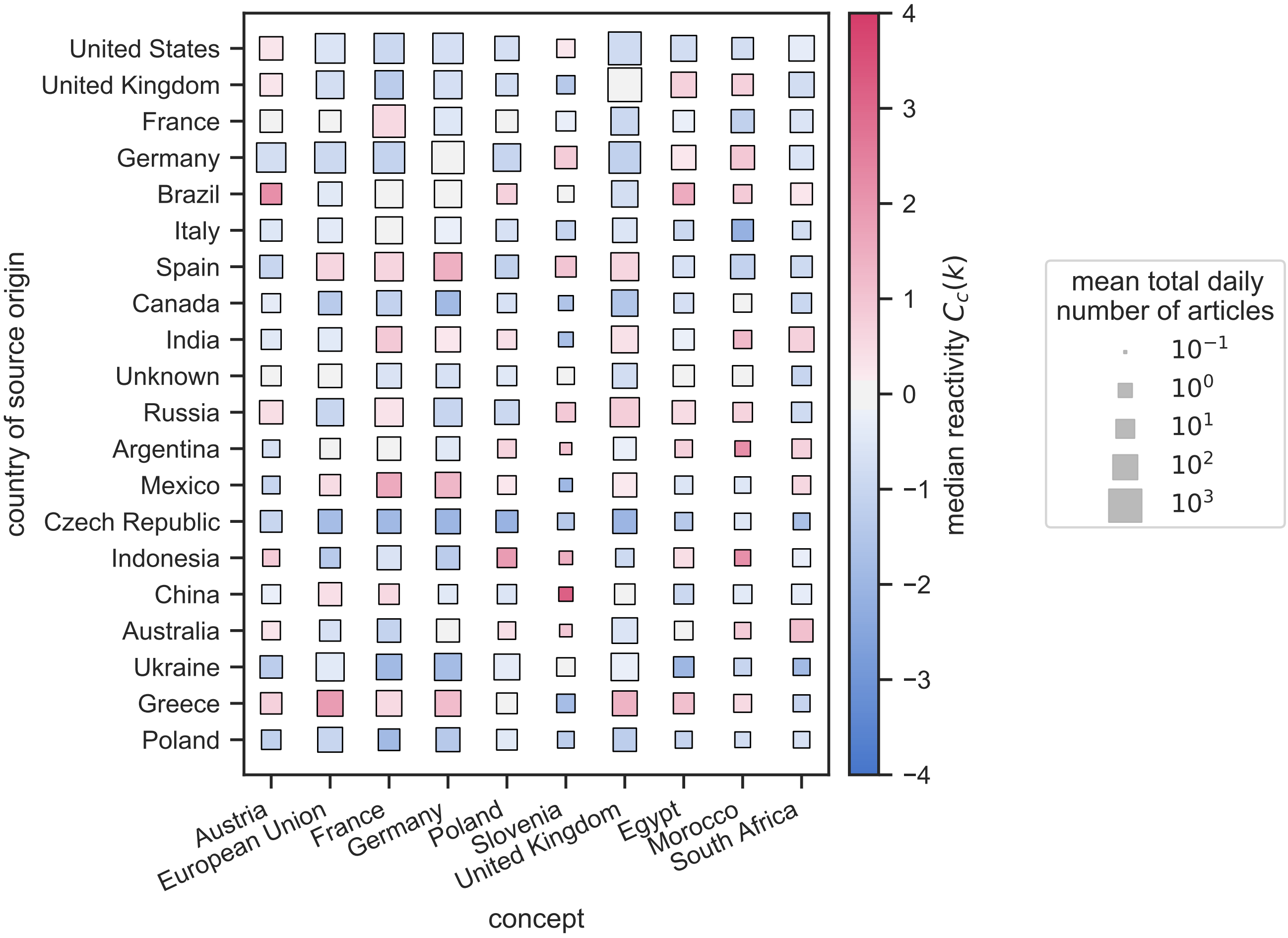

Figure S12. **The median reactivity $C_c(k)$ of the reactivity for all sources from country c for keywords k related to concepts related to selected African and European countries.** The square size is proportional to the total mean daily number of articles published by sources from country c on topic k; color represents the median reactivity $C_c(k) = \langle RA_s(k) \rangle_{s \in \mathscr{S}_c}$ of sources from the country c. Missing squares indicate that there were no publishers from a country that published at least 36 articles on a topic in our dataset. Red symbols correspond to topics that were reactively discussed in the country in 2018.

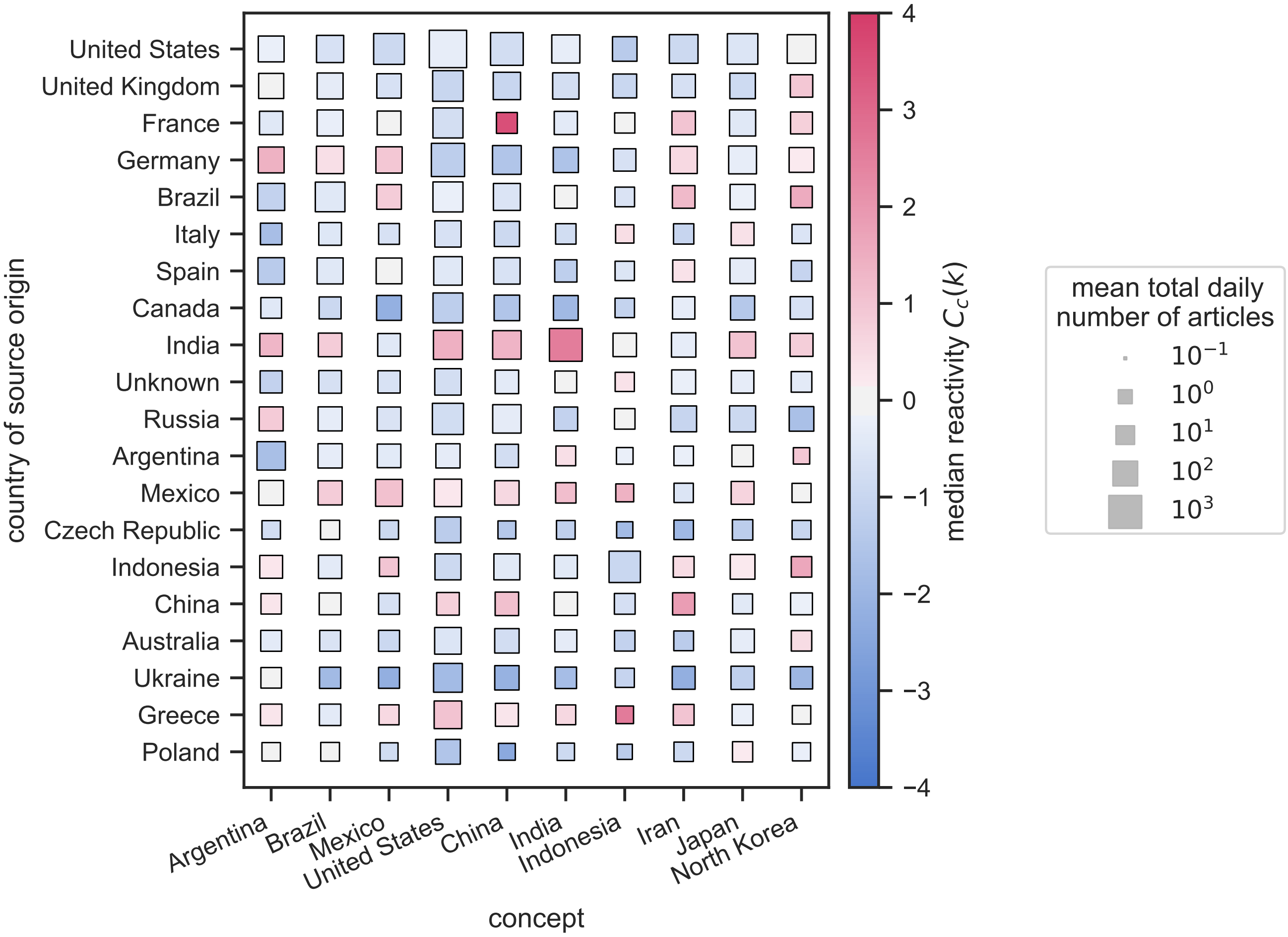

Figure S13. **The median reactivity $C_c(k)$ of the reactivity for all sources from country c for keywords k related to concepts related to selected American and Asian countries.** Square size is proportional to the total mean daily number of articles published by sources from country c on topic k; color represents a median reactivity $C_c(k) = \langle RA_s(k) \rangle_{s \in \mathscr{S}_c}$ of sources from country c. Missing squares indicate that there were no publishers from a country that published at least 36 articles on a topic in our dataset. Red symbols correspond to topics that were reactively discussed in the country in 2018.

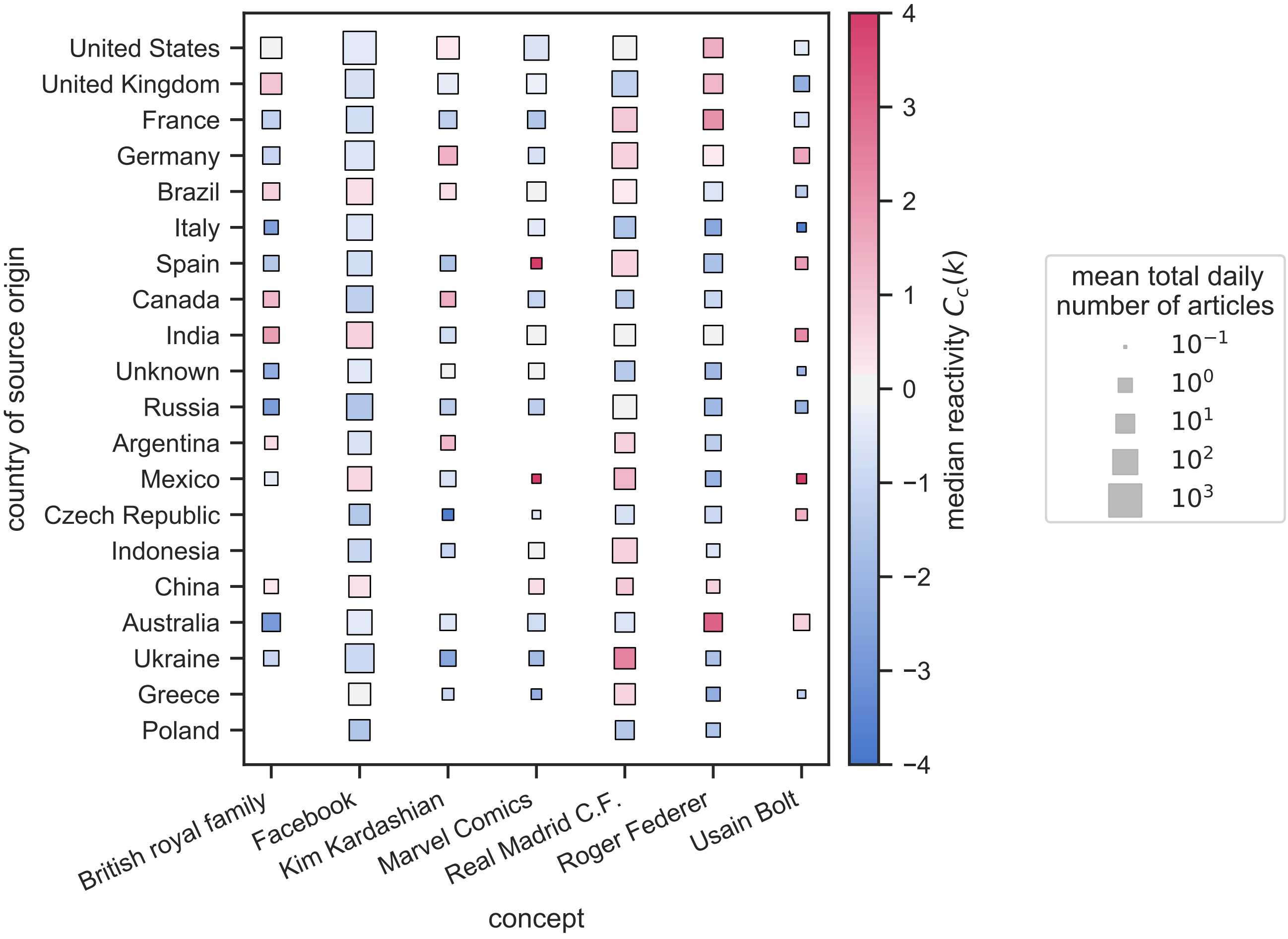

Figure S14. The median reactivity $C_c(k)$ of the reactivity for all sources from country c for keywords k related to selected concepts related to popular culture. Square size is proportional to the total mean daily number of articles published by sources from country c on topic k; color represents the median reactivity $C_c(k) = \langle RA_s(k) \rangle_{s \in \mathscr{S}_c}$ of sources from the country c. Missing squares indicate that there were no publishers from a country that published at least 36 articles on a topic in our dataset. Red symbols correspond to topics that were reactively discussed in the country in 2018.

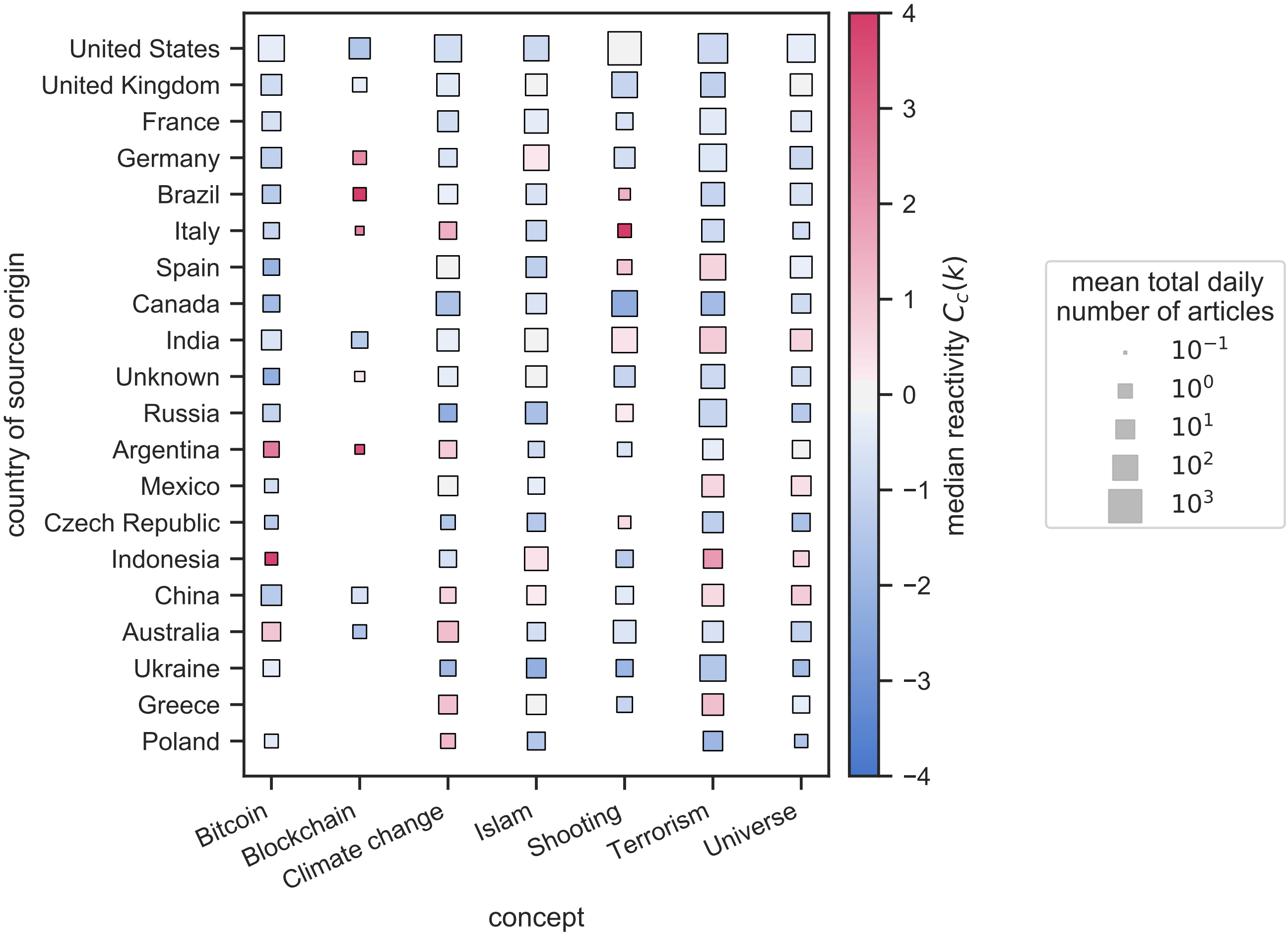

Figure S15. A median reactivity $C_c(k)$ of the reactivity for all sources from country c for keywords k related to other concepts selected for the study. Square size is proportional to the total mean daily number of articles published by sources from country c on topic k; color represents a median reactivity $C_c(k) = \langle RA_s(k) \rangle_{s \in \mathscr{S}_c}$ of sources from country c. Missing squares indicate that there were no publishers from a country that published at least 36 articles on a topic in our dataset. Red symbols correspond to topics that were reactively discussed in the country in 2018.

6 Measure comparisons

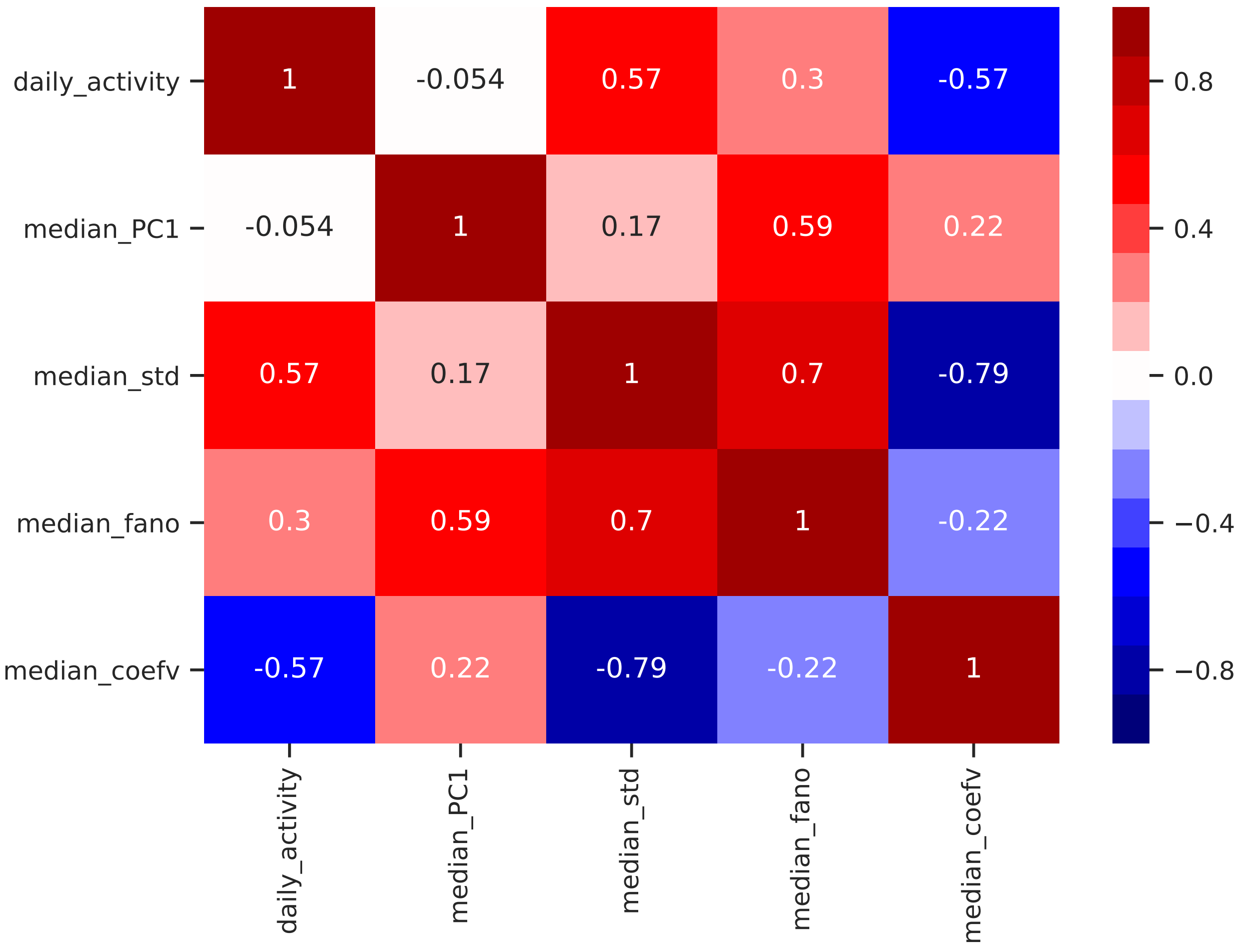

Figure S16. **Spearman correlation matrix of variability measures.** The median value of each measure was calculated for each *country-keyword* combination over all publishers from a country. Correlations were calculated over all possible *country-keyword* combinations. *daily activity* – total number of articles, *median RA* - median reactivity, *median std* – median of standard deviation (σ), *median fano* – median of the Fano factor ($\frac{\sigma^2}{\mu}$), *median coefv* – mean coefficient of variation ($\frac{\sigma}{\mu}$).

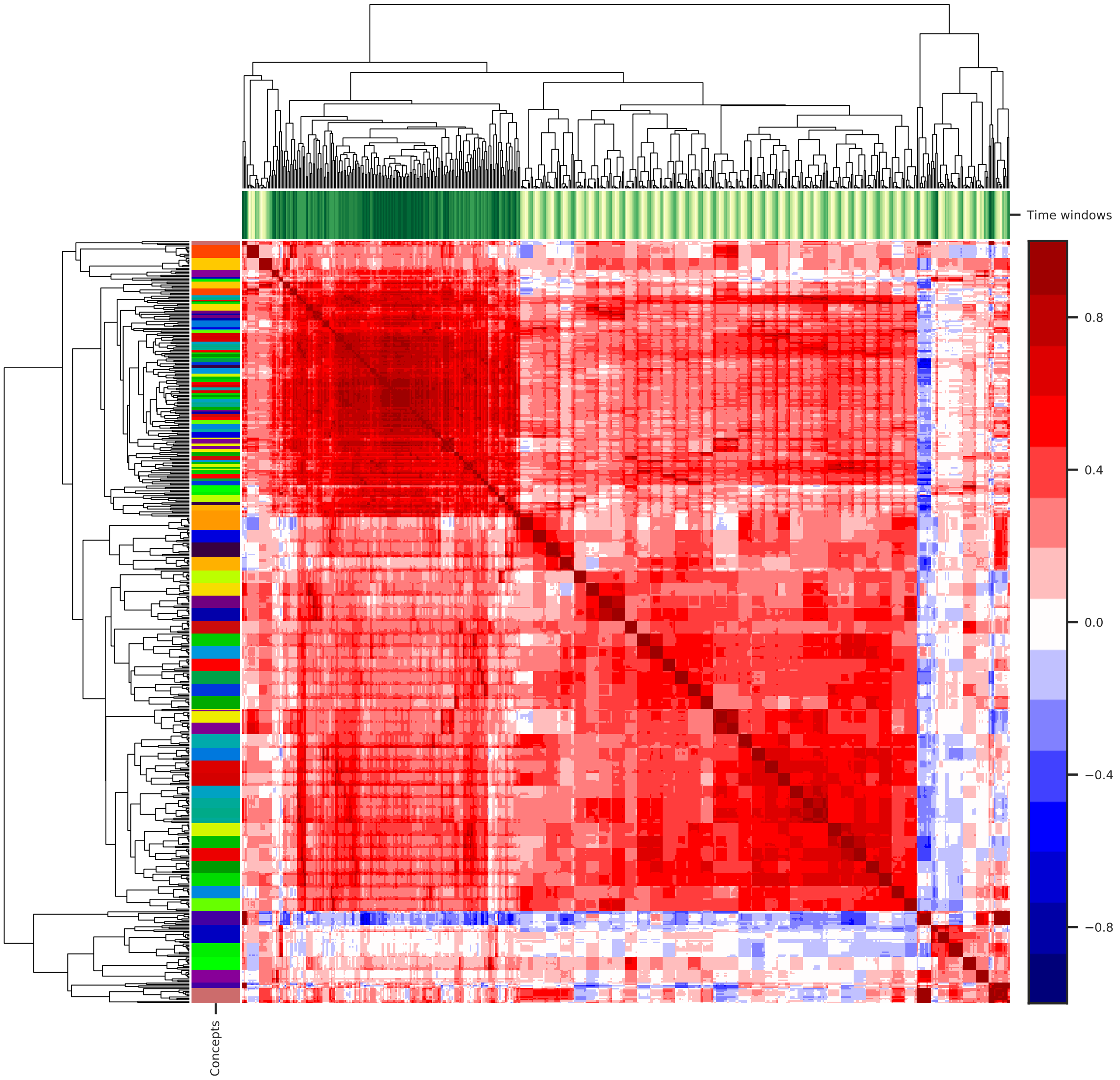

Figure S17. Hierarchical clustering of the correlation matrix of coefficients of variation for all concepts and time window sizes. Each row/column stands for one (k, Δ) combination. A color axis for the correlation matrix is on the right hand side. Additional color labels on top of the matrix represent time window size Δ (lightest – 5 minutes, darkest – 30 days); color labels on the left side of the matrix represent different keywords k. Residuals for short (below 1 hour) and long (over 1 hour) time windows are clustered together; inside the two clusters, residuals for the most of keywords tend to be close to each other.

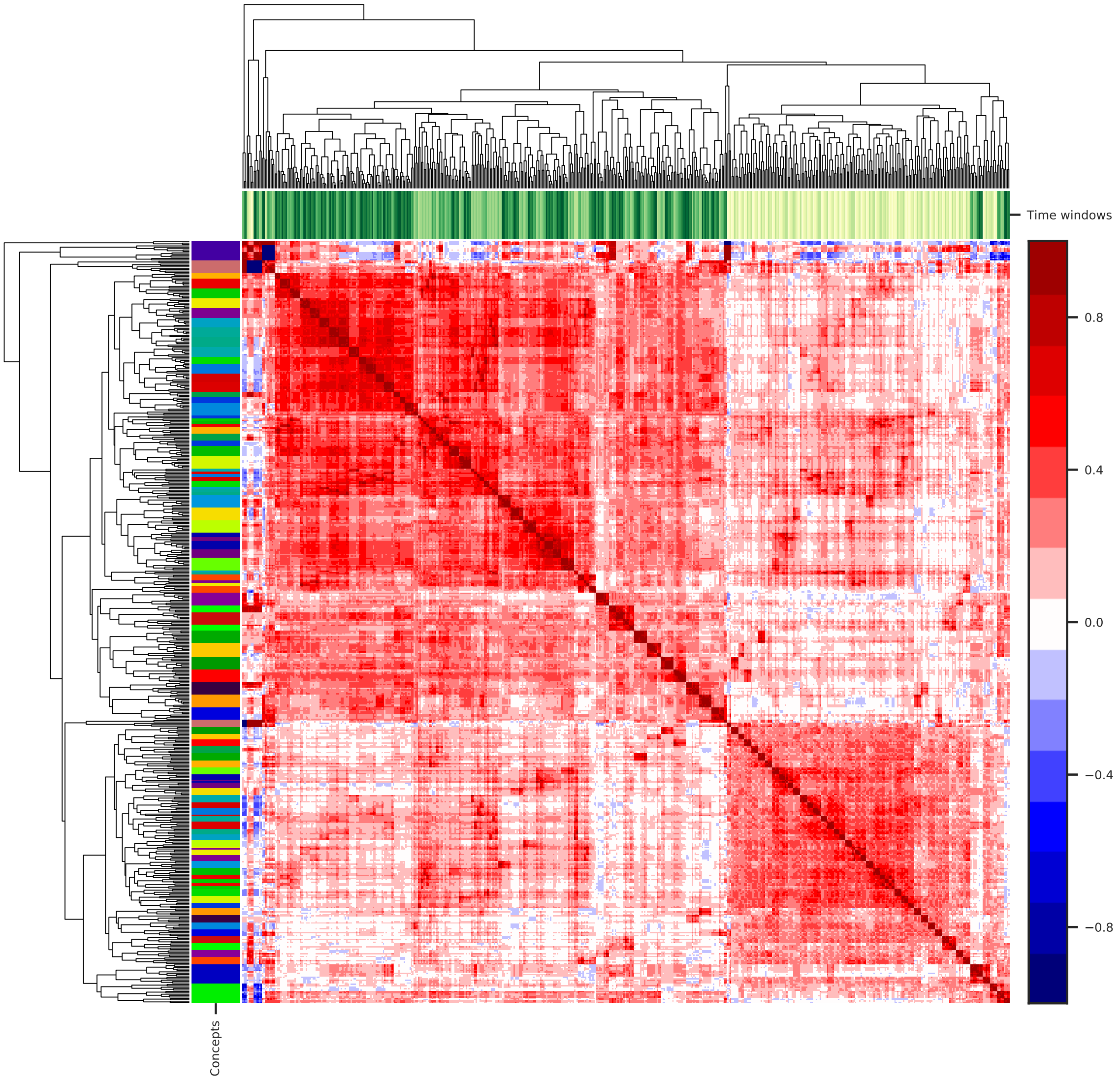

Figure S18. Hierarchical clustering of a correlation matrix of Fano factors for all concepts and time window sizes. Each row/column stands for one (k,Δ) combination. A color axis for the correlation matrix is on the right hand side. Additional color labels on top of the matrix represent a time window size Δ (lightest – 5 minutes, darkest – 30 days); color labels on the left side of the matrix represent different keywords k. Residuals for short (below 1 hour) and long (over 1 hour) time windows are clustered together; inside the two clusters, residuals for the most of keywords tend to be close to each other.

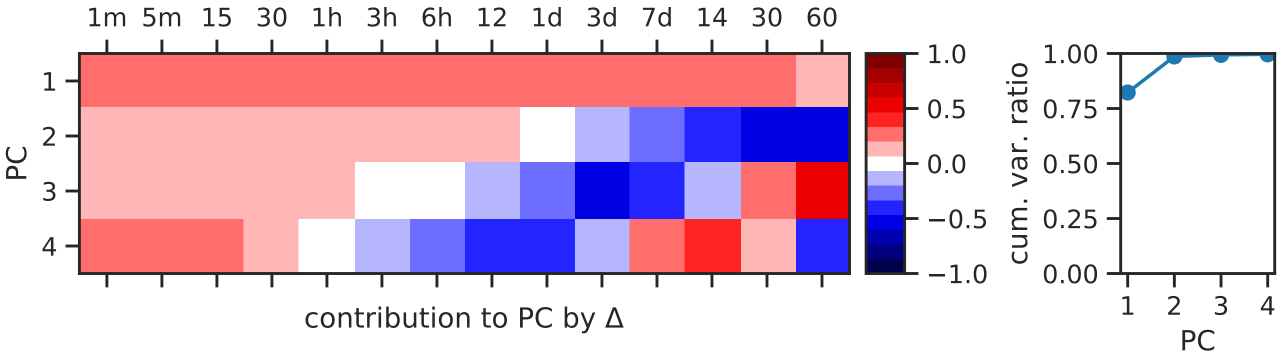

Figure S19. Principal Component Analysis of coefficient of variation by time window size for keyword *European Union* (clean data). (left) Contributions of corrections for different Δ to first four PCs (matrix Π). (right) A cumulative explained variance ratio for first four PCs.

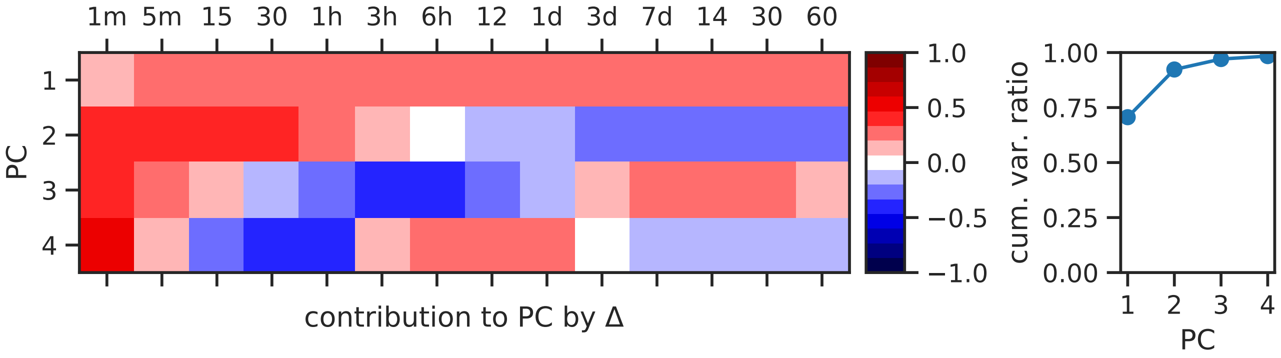

Figure S20. Principal Component Analysis of Fano factor by time window size for keyword *European Union* (clean data). (left) Contributions of corrections for different Δ to first four PCs (matrix Π). (right) A cumulative explained variance ratio for first four PCs.

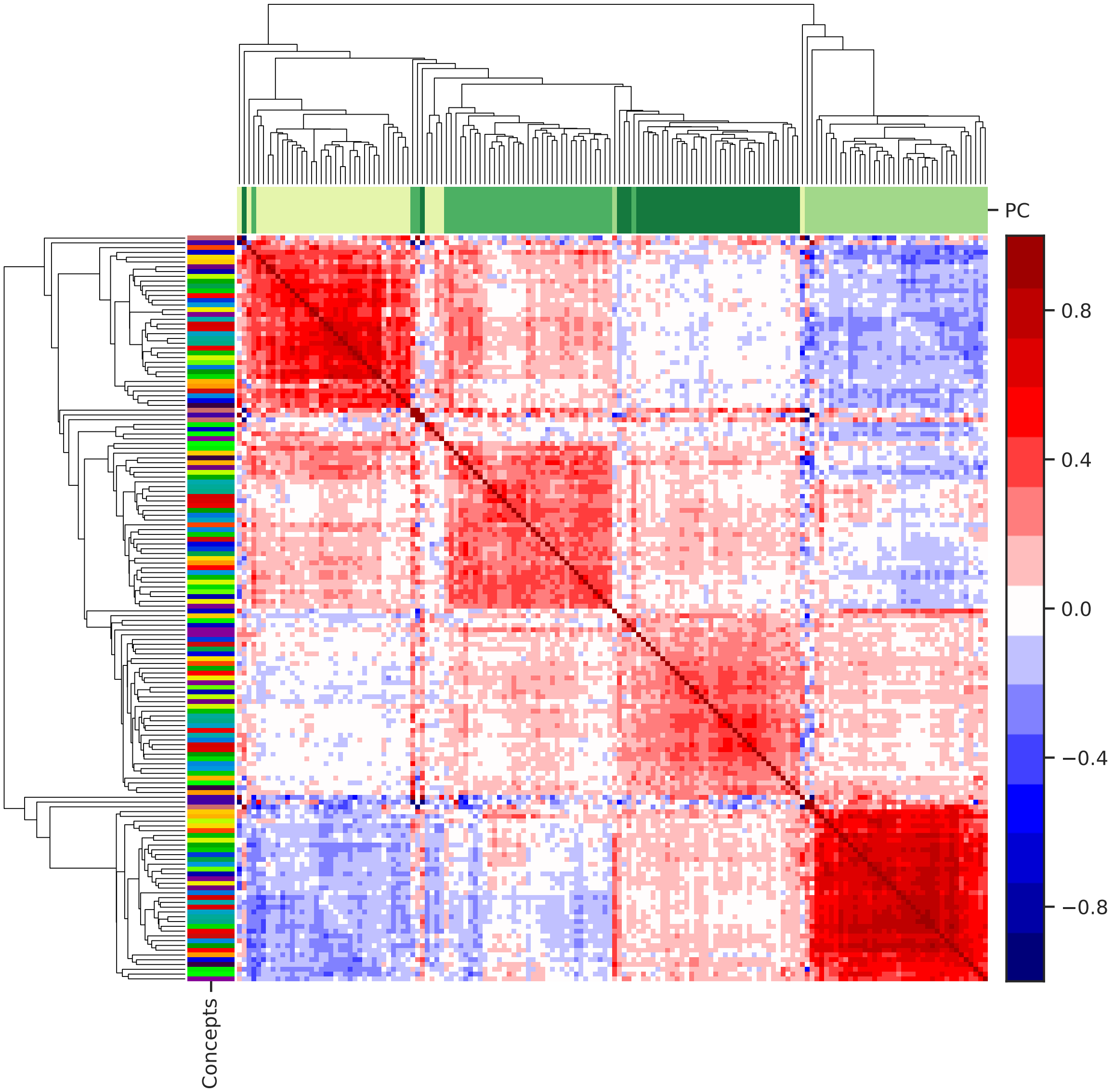

Figure S21. Hierarchical clustering of the correlation matrix of coefficient of variation PCs for all concepts. Each row/column stands for one (k, p) combination. A color axis for the correlation matrix is on the right hand side. Additional color labels on top of the matrix represent a number of PC p (lightest – 1st PC, darkest – 4th PC); color labels on the left side of the matrix represent different keywords k. PCs of similar order are clustered together.

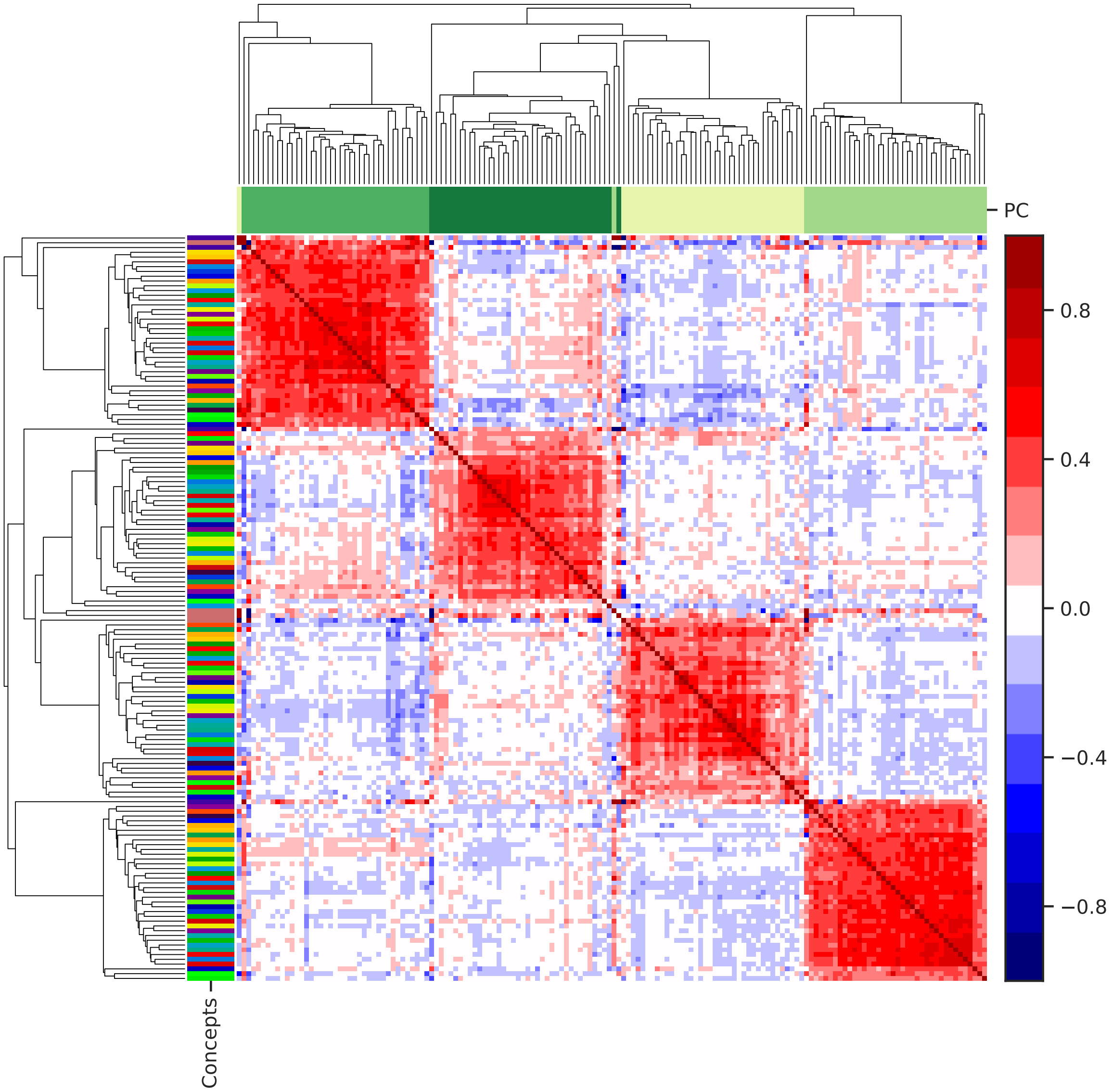

Figure S22. Hierarchical clustering of the correlation matrix of Fano factor PCs for all concepts. Each row/column stands for one (k, p) combination. A color axis for the correlation matrix is on the right hand side. Additional color labels on top of the matrix represent a number of PC p (lightest – 1st PC, darkest – 4th PC); color labels on the left side of the matrix represent different keywords k. PCs of similar order are clustered together.

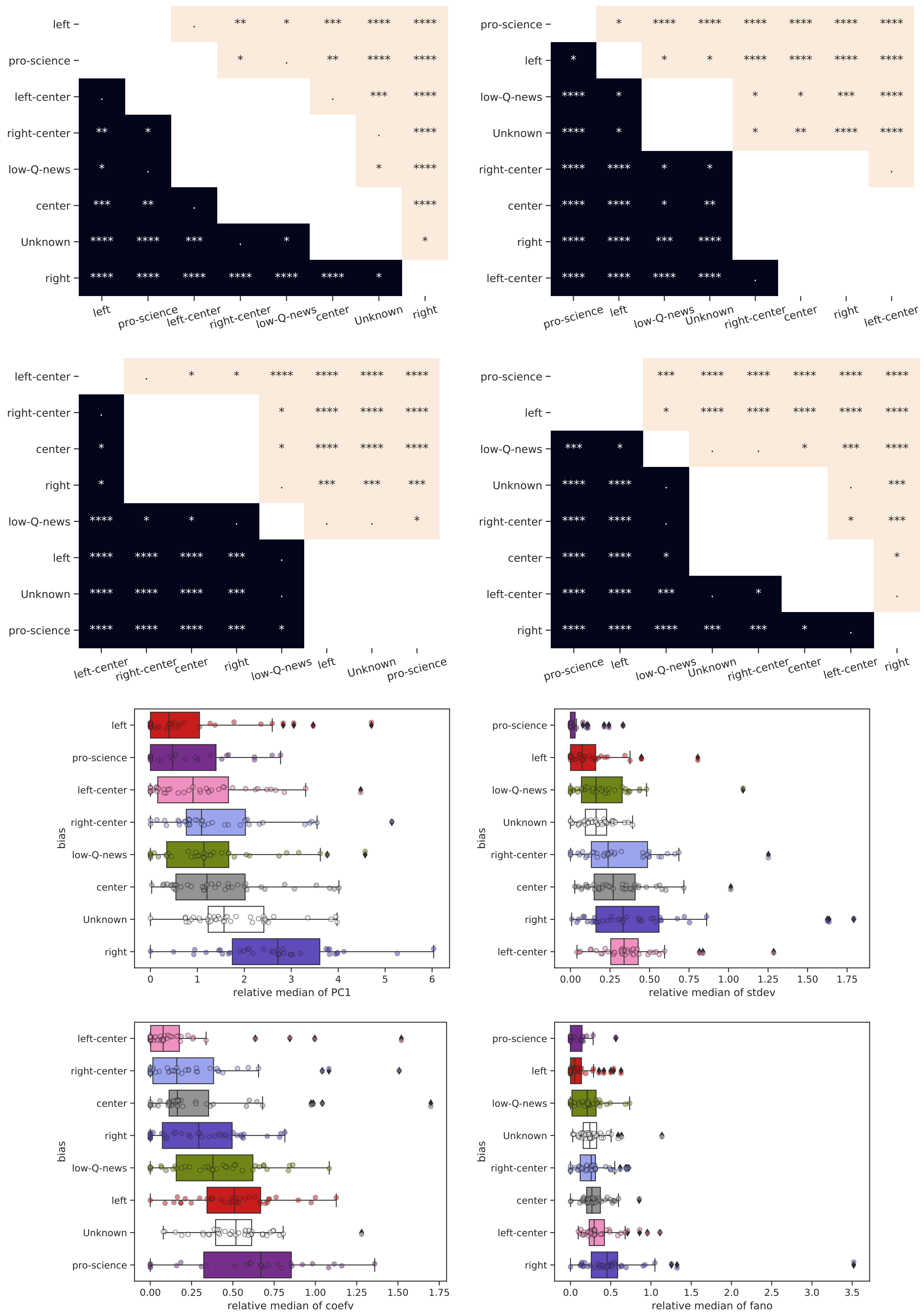

Figure S23. **Comparison of relative median of measures between bias groups for all keywords.**. top left – reactivity RA, top right – standard deviation σ, bottom left – coefficient of variation $\frac{\sigma}{\mu}$, bottom right – Fano factor $\frac{\sigma^2}{\mu}$. Top panels: median value of the measure for all keywords by political bias. Bottom panels: results of pairwise Dunn median tests with a two-step Benjamini-Krieger-Yekutieli FDR adjustment; for the rest of caption – see the main text.
